# Synergistic impact of ECAP and thermal treatments on the corrosion performance of AZ91 Mg alloy

**DOI:** 10.1371/journal.pone.0354359

**Published:** 2026-09-25

**Authors:** Gajanan M. Naik, M. Rajesh, S. Ramesh, Madhavi Magi, Shiv Pratap Singh Yadav, Ravi Kumar S. R, Gajanan Anne, Priyaranjan Sharma

**Affiliations:** 1 Department of Mechanical Engineering, RV Institute of Technology and Management, Bangalore, Karnataka, India; 2 Department of Industrial Engineering and Management, M S Ramaiah Institute of Technology, Bangalore, India; 3 School of Computer Science and Engineering, RV University, Bangalore, Karnataka, India; 4 Department of Mechanical Engineering, Jain College of Engineering, Belagavi, Karnataka, India; 5 Department of Mechanical Engineering, Nitte Meenakshi Institute of Technology, Nitte (Deemed to be University), Bengaluru, India; 6 Department of Mechanical Engineering, Dayananda Sagar College of Engineering, Bengaluru, Karnataka, India; 7 Manipal Institute of Technology, Manipal Academy of Higher Education, Manipal, India; 8 Department of Mechanical Engineering, Manipal University Jaipur, Jaipur, Rajasthan, India; Lovely Professional University, INDIA

## Abstract

This research examines the synergistic action of Equal-Channel-Angular-Pressing (ECAP) and heat treatments on the corrosion characteristics of AZ91 Mg alloy, a metal that is renowned for its mechanical and light weight properties but flawed by susceptibility to corrosion. The AZ91 alloy was ECAP for two and four passes to reduce its microstructure and afterwards, followed by the treatment of annealing and aging at the temperature of 523 K, 623 K and 723 K with treatment times of 6 and 12 hours. In order to investigate grain structure and secondary phase distribution, microstructural analysis was carried out using energy dispersive X-ray spectroscopy (EDX), optical microscopy (OM), and scanning electron microscopy (SEM). Potentiodynamic polarization experiments in a 3.5 wt. % NaCl environment were performed to assess corrosion behavior and found that the ECAP and subsequent heat treatments greatly improved corrosion resistance. Tests found that a combination of ECAP followed by annealing at 723 K for 12 hours produced a uniform distribution of secondary phases, with resultant smaller corrosion current densities and better electrochemical stability. The results show that the coupled processing methods successfully enhance the corrosion performance of AZ91 Mg alloy, which offers significant findings for its use in corrosive environments.

## 1. Introduction

Magnesium alloys, especially the AZ91 alloy, have been of great interest in materials science due to their light weight, high mechanical properties, and wide range of possible applications in industries like automotive and aviation [1–3]. Yet, the corrosion propensity of magnesium alloys in harsh environments presents a major drawback that restricts their full exploitation [4]. Since corrosion behavior is strongly governed by microstructural features, various processing routes have been explored to tailor the microstructure for improved performance. One of the most promising approaches to enhance the mechanical characteristics and corrosion resistance of magnesium alloys is Equal-Channel-Angular-Pressing (ECAP) [5,6]. ECAP is a severe plastic deformation technique that refines the microstructure by creating a high density of dislocations and grain boundaries, contributing to strength was significantly enhanced after ECAP, while ductility showed either slight improvement or marginal reduction depending on processing condition. Microstructural evolution through ECAP has the potential to strongly impact the corrosion performance of magnesium alloys [7,8]. Aside from ECAP, thermal processes like annealing and aging may also change the microstructure of magnesium alloys, enhancing the precipitation of secondary phases that can improve corrosion resistance. The interaction between ECAP and thermal processing is important in optimizing microstructure for better corrosion performance. Research has identified that the morphology and distribution of secondary phases like β-Mg_17_Al_12_ are responsible for influencing the corrosion behavior of AZ91 magnesium alloys. Although there have been improvements in the knowledge of how ECAP and thermal treatments affect the mechanical characteristics of magnesium alloys, there still exist gaps in the literature about how they act together to influence the corrosion performance [9,10].

Recent investigations indicate that ECAP followed by aging significantly alters both the microstructure and corrosion characteristics of AZ alloys [10–13]. Immersion tests in NaCl solutions demonstrated that ECAP enhanced corrosion resistance by refining grains and reducing the fraction and continuity of β-phase particles, thereby mitigating galvanic coupling. Nevertheless, subsequent aging promoted the precipitation of fine β-Mg_17_Al_12_ particles, which increased micro-galvanic activity and partially offset the beneficial effect of ECAP, although corrosion resistance remained superior to the as-cast alloy [11–13]. Similarly, Borko et al. observed that ECAP produced finer grains with a more homogeneous distribution of intermetallic phases. Their potentiodynamic polarization studies revealed a positive shift in corrosion potential (Ecorr), signifying a slight improvement in corrosion resistance compared with the as-cast alloy; however, the enhancement was limited due to the intrinsic reactivity of magnesium in chloride-containing media [14]. Complementary findings further revealed that combining ECAP with prior or post-aging treatments offers an effective strategy for optimizing the mechanical response. Specifically, prior aging promotes dynamic recrystallization during ECAP, generating fine, equiaxed grains with dispersed precipitates that enhance ductility, whereas post-aging favors precipitation hardening, thereby improving yield strength. These results underscore that both corrosion resistance and mechanical performance in AZ91 alloys can be flexibly tailored through the careful sequencing of ECAP and thermal treatments [15].

The purpose of this research is to examine the synergistic effects of ECAP and thermal treatments on the corrosion resistance and microstructure of the AZ91 Mg alloy. Through systematic investigation of microstructural development and corrosion properties under different conditions of processing, this work aims to offer insights into optimizing magnesium alloys for increased performance under corrosive conditions [8,16–19]. Recent efforts in the field have primarily focused on advancements in severe plastic deformation (SPD) and heat treatment strategies. For instance, Yang et al. reported global advancement in ECAP and corrosion studies [11], while Elambharathi et al. provided a comprehensive review of emerging heat-treatment approaches for Mg alloys [20]. However, comparatively fewer investigations have explored the integrated role of ECAP with annealing or aging in AZ91 alloys. The present work addresses this gap by establishing a systematic framework that combines SPD and thermal processing, thereby elucidating quantitative correlations between microstructure, corrosion resistance, and mechanical properties.

This work systematically demonstrates the synergistic effects of ECAP followed by annealing/aging on the AZ91 magnesium alloy. Unlike previous studies that examined either SPD or thermal treatment in isolation, this research highlights how the combined processing leads to grain refinement, homogeneous redistribution of the β-Mg_17_Al_12_ phase, improved mechanical properties and significantly enhanced corrosion resistance. Using advanced characterization techniques and electrochemical testing, the study provides detailed structure–property–corrosion correlations that have not been previously reported for AZ91. These findings offer valuable insights for optimizing processing prameters, supporting the broader application of magnesium alloys in corrosive environments.

## 2. Materials and experimental procedure

The AZ91 magnesium alloy used in this study was supplied in wrought form with a nominal composition of ~9% aluminum and ~1% zinc. Its chemical composition was confirmed by optical emission spectrometry to ensure compliance with standard specifications. Samples were sectioned from the ingots and prepared for subsequent processing. ECAP was performed using a 90° channel die, route R (reversal/inverted), a pressing speed of 1 mm/s, and MoS_2_ lubrication. Billets with dimensions of 16 mm in diameter and 80 mm in length were processed at 598K to enhance plasticity and suppress cracking. These two deformation levels were investigated: two passes (ECAP-2P) and four passes (ECAP-4P), with cooling to room temperature between passes. These two levels were chosen because preliminary trials and previous reports indicated that more than four passes in AZ91 often result in cracking and excessive die wear due to the alloy’s limited ductility at the selected processing temperature [5,6]. Two passes provided the initial refinement of coarse grains, while four passes promoted extensive grain size reduction and homogeneous redistribution of the β-Mg_17_Al_12_ phase. This selection allowed us to capture both intermediate and advanced deformation states without causing process instability, thereby enabling reliable evaluation of microstructural trends and corrosion behavior.

Following ECAP, the samples underwent thermal treatments, including annealing and aging at 523 K, 623 K, and 723 K for durations of 6 and 12 hours. These treatments aimed to enhance precipitation and redistribute secondary phases. Annealing was limited to 723 K because this temperature is close to the incipient melting point of the β-Mg_17_Al_12_ phase in AZ91, beyond which microstructural coarsening and loss of mechanical properties can occur [21]. Therefore, the range of 523–723 K was selected as a practical window to balance precipitation, homogenization, and phase stability while avoiding melting-related damage. Specifically, 523 K corresponds to the stage of initial precipitation and clustering, 623 K represents intermediate coarsening and redistribution, and 723 K approaches near-homogenization of the β-Mg_17_Al_12_ phase. This interval allowed systematic evaluation of the trade-off between corrosion stability and precipitation strengthening.

Microstructural characterization involved Optical Microscope (OM) to reveal grain structures, Scanning Electron Microscope (SEM) to study the morphology and distribution of secondary phases, and energy-dispersive X-ray spectroscopy (EDS) for elemental analysis. Sample preparation followed standard metallographic procedures, including grinding, polishing, and etching to ensure clear visualization of microstructural features. The values of average grain size were obtained based on several representative grains analyzed by image analysis software, and their values are reported in mean ± standard deviation form.

A three-electrode electrochemical setup was used to assess corrosion behavior using potentiodynamic polarization in a 3.5 weight percent NaCl solution. The reference electrode was a saturated calomel electrode, and the counter electrode was a platinum electrode. Before testing, all samples were stabilized at open-circuit potential for 30 minutes. Tafel extrapolation was used to calculate corrosion parameters, including corrosion potential (Ecorr) and corrosion current density (Icorr). All electrochemical tests were performed in triplicate under identical conditions, and the reported values represent averaged results. The experimental deviation was within acceptable limits for corrosion testing of magnesium alloys. SEM was used to analyze the corroded samples’ surface morphology. Elemental composition and corrosion product formation were further investigated through EDS. The corrosion rate for the corresponding samples was calculated using Equation (1).


$$\begin{array}{c} \text{CR}(\text{mm}/\text{y})\;=\;\text{3}.\text{27 x }{\text{10}}^{-\text{3}}\;\frac{i_{corr}\;X\;A}{n\;\rho } \end{array}$$ (1)

Where: CR = Corrosion rate in mils per year (mpy) (later converted to mm/year), A = Molar mass (for magnesium 24.3 g/mol), icorr = Corrosion current density (µA/cm²), n = Valence of metal (for Mg n = 2), ρ (rho) = Density (1.74 g/cm³)

## 3. Results and discussion

### 3.1. Comparison of Microstructure in as-received and ECAPed AZ91 Mg Alloy

**Fig 1:**
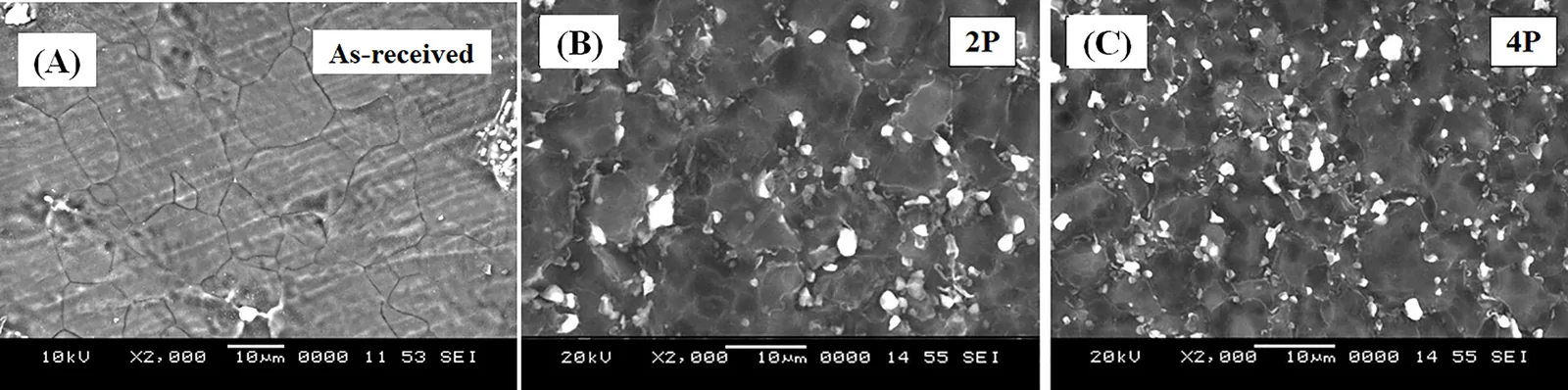
SEM micrographs of AZ91 magnesium alloy: (A) as-received condition (average grain size: 58.69 µm), (B) ECAP-2P (30.86 µm), and (C) ECAP-4P (7.58 µm), demonstrating progressive grain refinement after ECAP processing.

Fig 1 shows the SEM microstructures of the AZ91 magnesium alloy in its as-received condition and after ECAP at 598 K with two different pass numbers: two passes (2P) and four passes (4P). The micrograph of the as-received AZ91 alloy (Fig 1A) reveals a coarse-grained structure characterized by large, irregular grains typical of wrought magnesium alloys. The results of quantitative grain size determination showed that the mean grain size of the untreated AZ91 alloy was about 58.69 µm, whereas it was reduced to 30.86 µm for AZ91 alloy subjected to two passes of ECAP (ECAP-2P) and 7.58 µm for the AZ91 alloy subjected to four passes of ECAP (ECAP-4P) are presented in Table 1. Secondary phase regions predominantly observed near grain boundaries, as indicated in Fig 1(A). These features are characteristic of the β-Mg_17_Al_12_ eutectic/secondary phase distribution commonly reported in AZ91 magnesium alloys. This initial microstructure contains a high fraction of secondary phases, primarily β-Mg_17_Al_12_, which are concentrated along grain boundaries. Such segregation can significantly influence mechanical properties and corrosion behavior by promoting galvanic coupling at these interfaces. The heterogeneous distribution of phases and coarse grain structure result in non-uniform mechanical performance and reduced corrosion resistance, similar observation was reported in literature [9,17].

**Table 1:** Grain size variation of AZ91 Mg alloy under ECAP processing and annealing treatments.

Annealing Temperature	Annealing Time (h)	Grain Size (µm)		
		As-received	ECAP-2P	ECAP-4P
Room Temp.	0	58.69	30.86	7.58
523K	6	59.10	31.45	7.82
	12	59.72	32.08	8.05
623K	6	59.95	32.76	8.34
	12	60.38	33.41	8.63
723K	6	60.12	34.18	8.06
	12	60.92	35.04	8.98

After ECAP processing at 598 K, pronounced microstructural refinement is observed, as illustrated in Fig 1B and 1C for the two-pass and four-pass conditions, respectively. The ECAP process imposes severe plastic deformation, generating a high density of dislocations and activating dynamic recrystallization, which refines the grain structure and leads to a more uniform grain size as widely reported in ECAP-processed magnesium alloys [22]. Following two passes (2P), the AZ91 alloy exhibits significantly finer grains compared to the as-received state, while the four-pass (4P) specimen shows even greater grain refinement and improved microstructural uniformity, this claim is supported by reference [12]. Additionally, the secondary phases display a more continuous and evenly distributed morphology within the matrix, contrasting with the pronounced grain boundary segregation of the as-received alloy. This refined and homogeneous microstructure is expected to enhance mechanical strength and ductility, while significantly improving corrosion resistance by reducing localized galvanic coupling and promoting more uniform, stable corrosion behavior in the ECAP-processed AZ91 magnesium alloy.

### 3.2. Transformation of microstructure in as-received AZ91 Mg alloy after thermal treatments

**Fig 2:**
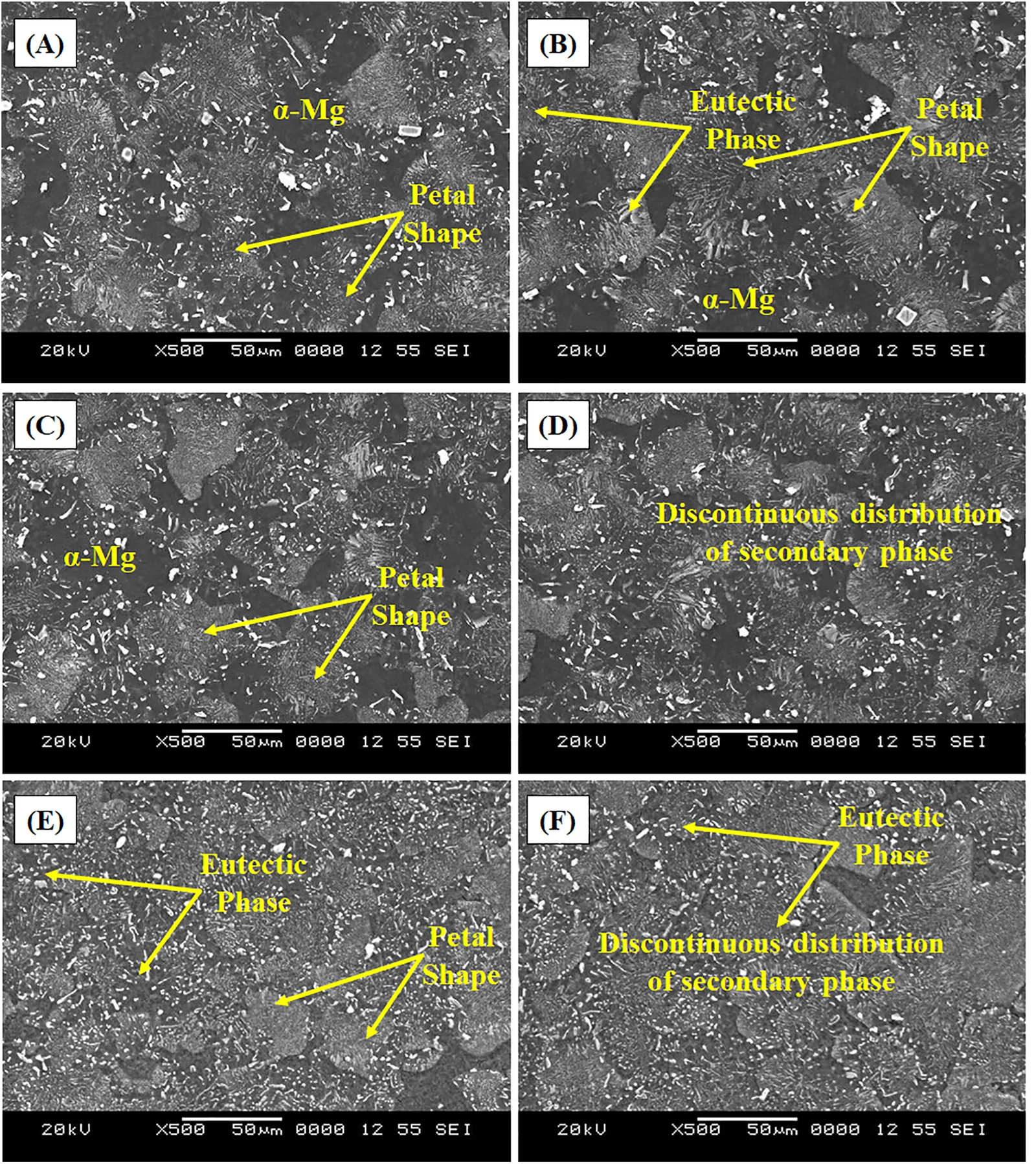
SEM micrographs of AZ91 Mg alloy following annealing at: (A) 523 K for 6 hours, (B) 523 K for 12 hours, (C) 623 K for 6 hours, (D) 623 K for 12 hours, (E) 723 K for 6 hours, and (F) 723 K for 12 hours.

The secondary phase β-Mg_17_Al_12_ and the α-Mg + β-Mg_17_Al_12_ eutectic areas are clearly identified in Figs 1 and 2 in order to make comparisons easier between the initial sample and the heat-treated samples. The eutectic phases, mainly located around grain boundaries are redistributed and slightly modified in morphology by increasing the annealing temperature and time. The microstructures of the as-received AZ91 alloy after annealing at 523 K, 623 K, and 723 K for 6 hours and 12 hours, are shown in the SEM micrographs of Fig 2. From the micrographs, the microstructures show the precipitation of secondary phases in the magnesium alloy, as well as the presence of a thin eutectic structure that indicates the presence of the α-Mg matrix and the β-Mg_17_Al_12_ intermetallic phase. Fig 2(A-F) show the microstructures of the alloy after annealing at different temperatures, ranging from 523 K to 723 K for 6 hours and 12 hours.

Annealing promoted a higher area fraction of the β-Mg_17_Al_12_ phase compared to the untreated alloy, which contributes positively to corrosion resistance. As illustrated in Fig 2 (A)–(F), both eutectic and discontinuous β-Mg_17_Al_12_ phases appear along the grain boundaries, displaying petal-like and eutectic structures that develop further with prolonged aging. Notably, the microstructure of the AZ91 alloy treated at 723 K showed a more refined and uniformly distributed secondary phase compared to samples annealed at 523 K and 623 K. Extending the annealing duration from 6 to 12 hours consistently increased the number of secondary phase precipitates across all temperature conditions. The interpretation of the microstructure changes after the heat treatments was done in consideration of the quantitative results obtained for the grain sizes presented in Table 1. In addition, the presence and morphology changes of the β-Mg_17_Al_12_ secondary phase seen from the SEM micrographs were further substantiated by the XRD findings.

**Fig 3:**
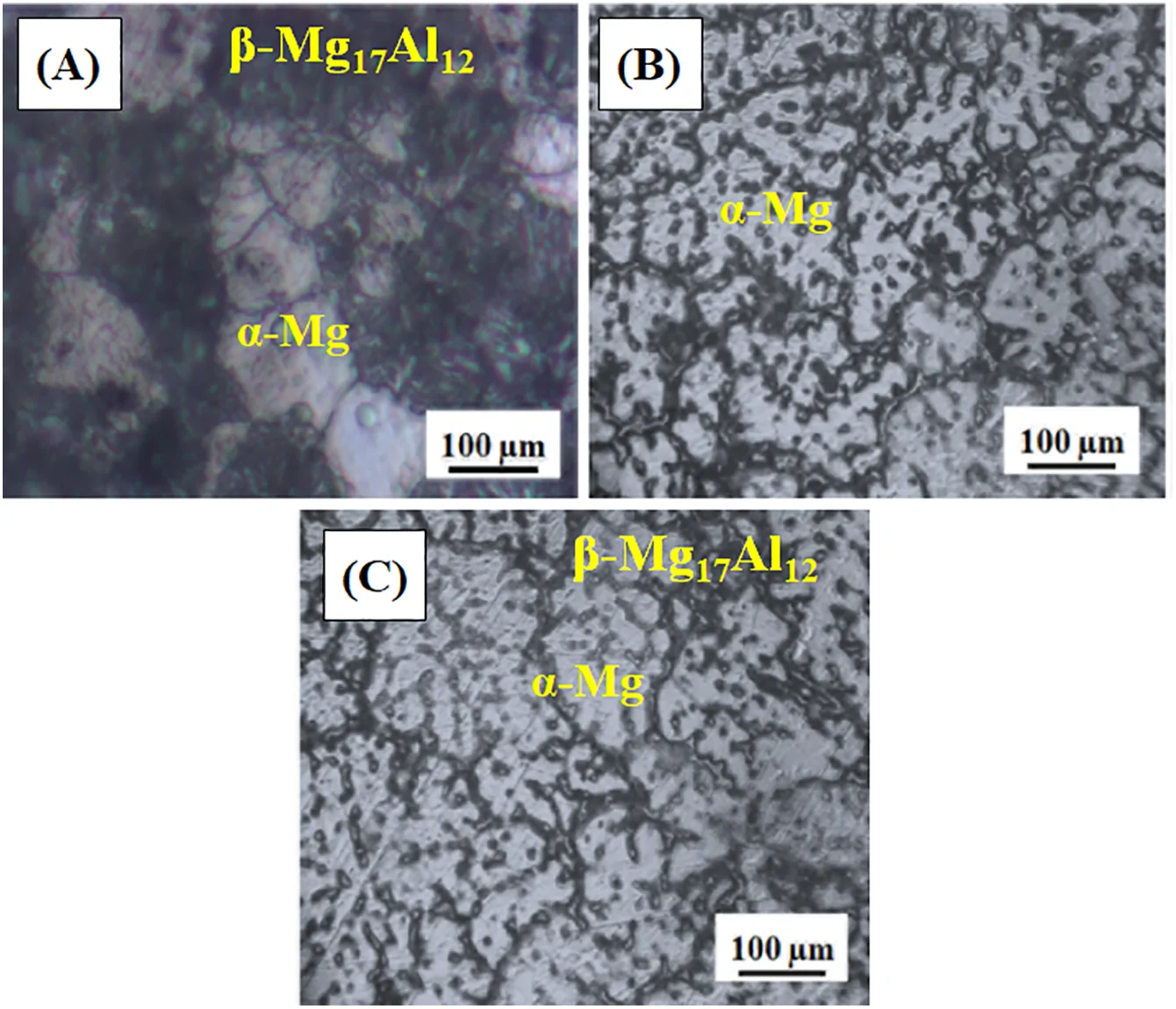
Optical Microscopy (OM) images showing the influence of temperature on grain growth in AZ91 magnesium alloy: (A) as-received condition (average grain size ≈ 58.69 µm), (B) annealed at 723 K for 6 hours (average grain size ≈ 60.12 µm), and (C) annealed at 723 K for 12 hours (average grain size ≈ 60.92 µm).

Grain growth after annealing was further investigated by Optical Microscopy (OM) as illustrated in Fig 3, in which grain size development under various annealing times is shown in Fig 3, with images labeled as (A) for the as-received condition, (B) for 723 K after 6 hours, and (C) for 723 K after 12 hours. This grain growth behavior is associated with improved corrosion resistance which is confirmed through electrochemical results presented in Table 2, as reported by Zhao et al. [21]. Measurements of average grain size, derived from the optical micrographs in Fig 3, confirm this effect: the as-received alloy exhibited an average grain size of approximately 58.69 µm (Fig 3 (A)). After annealing at 723 K for 6 hours, the average grain size increased slightly to around 60.12 µm (Fig 3 (B)), and further annealing for 12 hours at 723 K resulted in an average grain size of approximately 60.92 µm are presented in Table 1, demonstrating the influence of annealing time on grain growth.

**Table 2:** Summary of annealing and aging treatment effects on processed and unprocessed AZ91 Mg alloys.

Annealing Temperature	Annealing Time (h)	As-received (GS=58.69µm)			ECAP-2P(GS=30.86µm)			ECAP-4P (GS=7.58µm)		
		E_corr_ (V_SCE_)	I_corr_ (mA/cm^2^)	CR (mm/y)	E_corr_ (V_SCE_)	I_corr_ (mA/cm^2^)	CR (mm/y)	E_corr_ (V_SCE_)	I_corr_ (mA/cm^2^)	CR (mm/y)
Room Temp.	0	-1.539	0.2630	12.010	-1.548	0.0224	1.023	-1.488	0.0026	0.119
523K	6	-1.555	0.2610	11.919	-1.536	0.0128	0.585	-1.500	0.0028	0.128
	12	-1.548	0.1620	7.398	-1.510	0.0127	0.580	-1.512	0.0027	0.123
623K	6	-1.527	0.1200	5.480	-1.544	0.0129	0.589	-1.489	0.0021	0.096
	12	-1.507	0.0215	0.982	-1.510	0.00122	0.056	-1.408	0.0013	0.059
723K	6	-1.501	0.0235	1.073	-1.468	0.00120	0.055	-1.402	0.0012	0.055
	12	-1.489	0.0205	0.936	-1.428	0.00110	0.050	-1.400	0.0011	0.050

### 3.3. Microstructural analysis of AZ91 Mg alloys processed by ECAP

**Fig 4:**
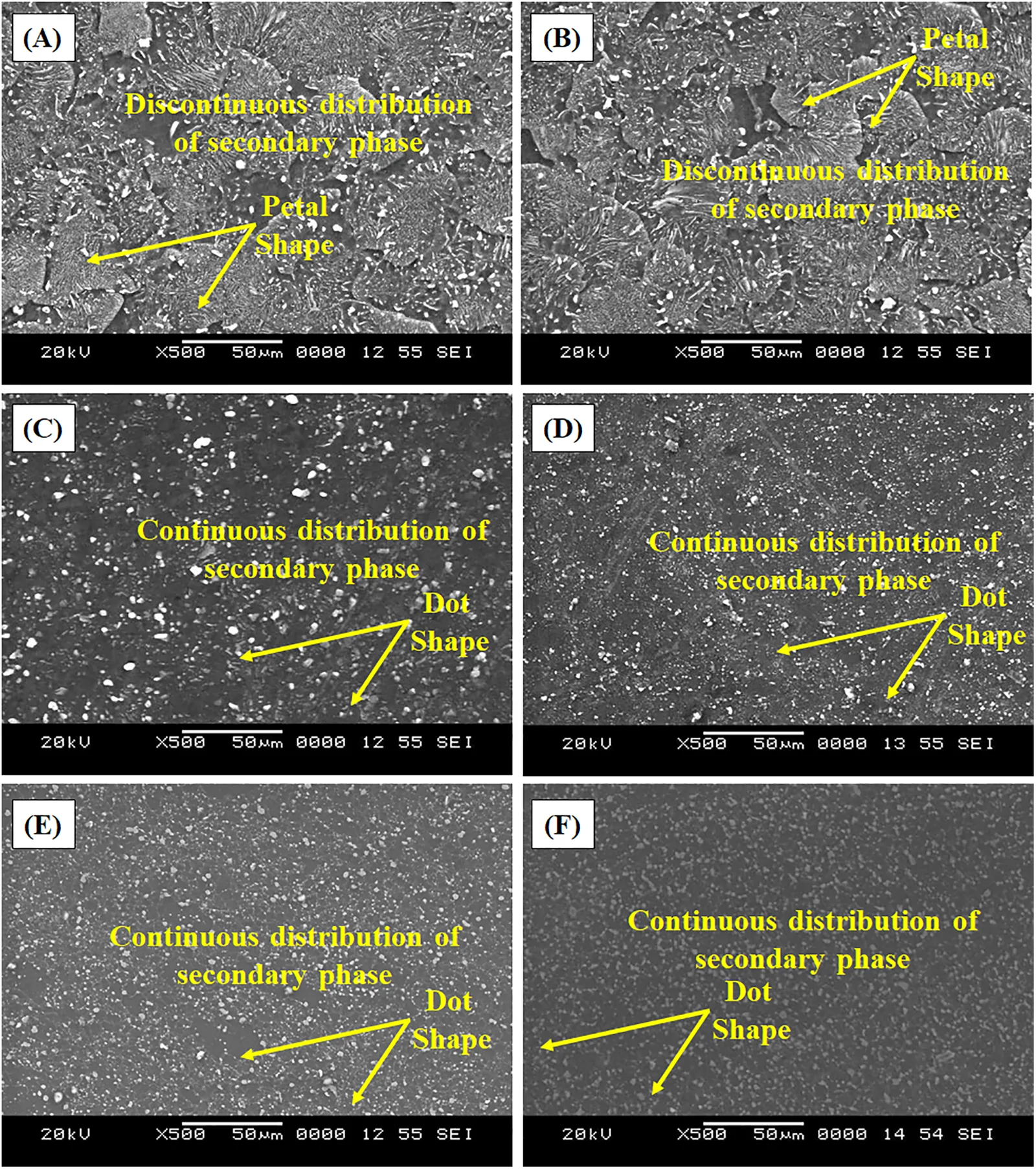
SEM micrographs of heat-treated ECAP-2P AZ91 magnesium alloy: (A) 523 K for 6 hours, (B) 523 K for 12 hours, (C) 623 K for 6 hours, (D) 623 K for 12 hours, (E) 723 K for 6 hours, and (F) 723 K for 12 hours.

**Fig 5:**
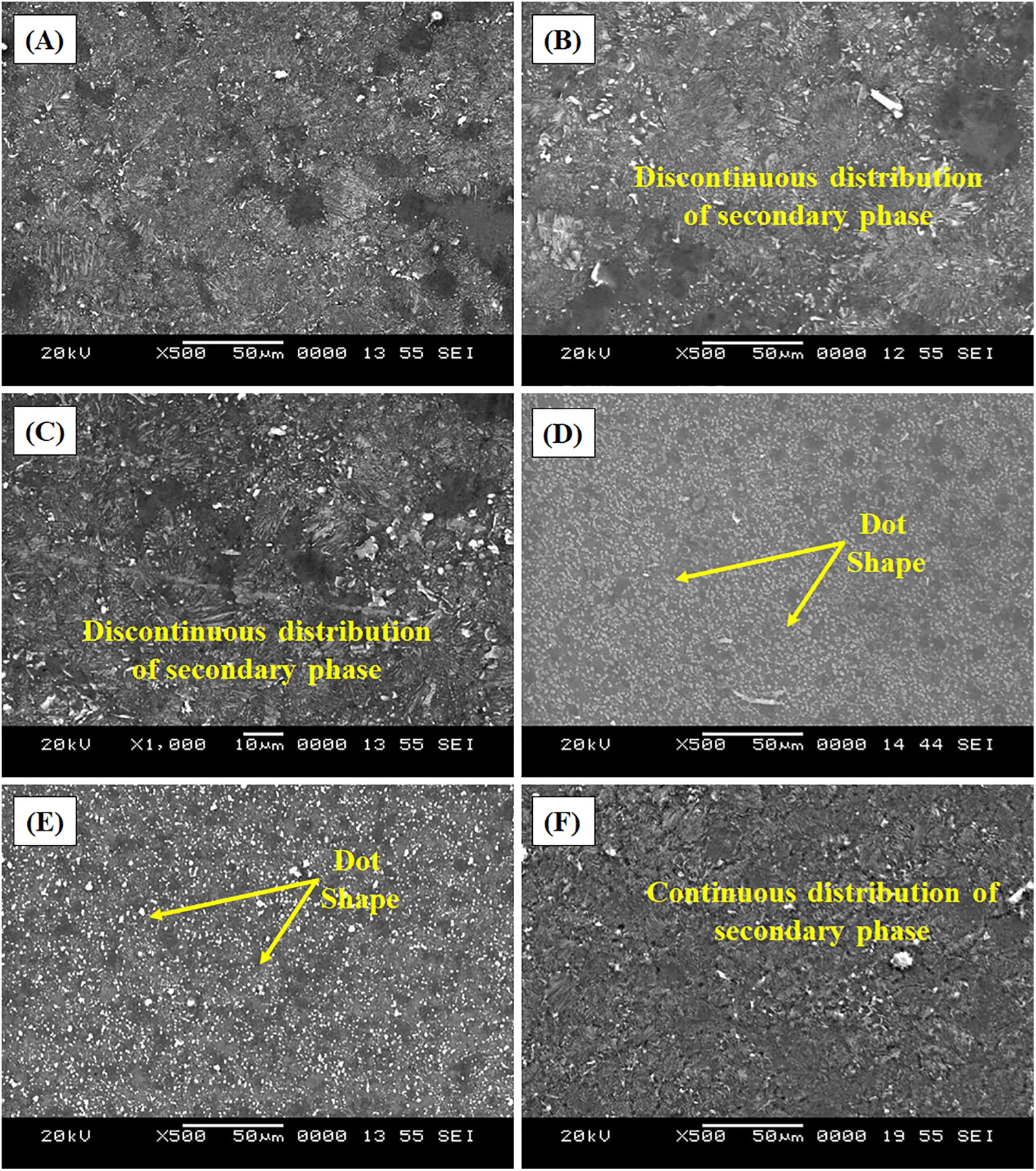
SEM micrographs of ECAP-4P AZ91 magnesium alloy after heat treatment: (A) 523 K for 6 hours, (B) 523 K for 12 hours, (C) 623 K for 6 hours, (D) 623 K for 12 hours, (E) 723 K for 6 hours, and (F) 723 K for 12 hours.

The microstructures of AZ91 magnesium alloy subjected to two-pass (ECAP-2P) and four-pass (ECAP-4P) processing, followed by heat treatments at 523 K, 623 K, and 723 K for durations of 6 and 12 hours, are presented in Figs 4 and 5, respectively. In comparison to the heat-treated as-received alloy (Fig 2), the ECAP-processed specimens display a noticeably more uniform and refined distribution of secondary phases across all annealing temperatures and holding times. This enhancement results primarily from the redistribution of phases achieved through the ECAP process, as evidenced by the marked differences in secondary phase arrangement before and after ECAP.

In the as-received AZ91 Mg alloy, secondary phases are predominantly segregated along grain boundaries. In contrast, the ECAPed alloys display a much more uniform distribution of these phases. This refined secondary phase distribution following ECAP is consistent with findings reported by Shahar et al. [23] and Huang et al. [24].

The redistribution of secondary phases in ECAPed AZ91 Mg alloys after heat treatment is illustrated in Fig 4 and Fig 5. Fig 4(A)–(F) presents the microstructures of ECAP-2P samples heat-treated at 523 K, 623 K, and 723 K for 6 and 12 hours. Notably, petal-shaped phases are observed in ECAP-2P samples treated at 523 K and 623 K for both time durations. In contrast, ECAP-2P samples treated at 723 K for 6 and 12 hours, as well as all ECAP-4P samples heat-treated at these three temperatures for both time intervals, the decrease in the discontinuous phases β-Mg_17_Al_12_ after heat treatment is indicative of gradual redistribution and partial dissolution of eutectics, which results in increased homogeneity of the microstructure. This stable and uniform phase distribution is evident in the SEM images in Figs 4(E), 4(F) and 5(A)–5(F), which contributes to the enhancement of corrosion resistance.

Additionally, comparing the AZ91 Mg alloy heat-treated at 523 K, 623 K, and 723 K for 6 hours and 12 hours reveals that the fraction of β-Mg_17_Al_12_ phases increases noticeably with longer heat treatment, resulting in a finer and more homogeneous distribution. These observations are in line with findings by Yongdong et al. [25] for Mg-Nd-Gd-Zn-Zr alloys.

**Fig 6:**
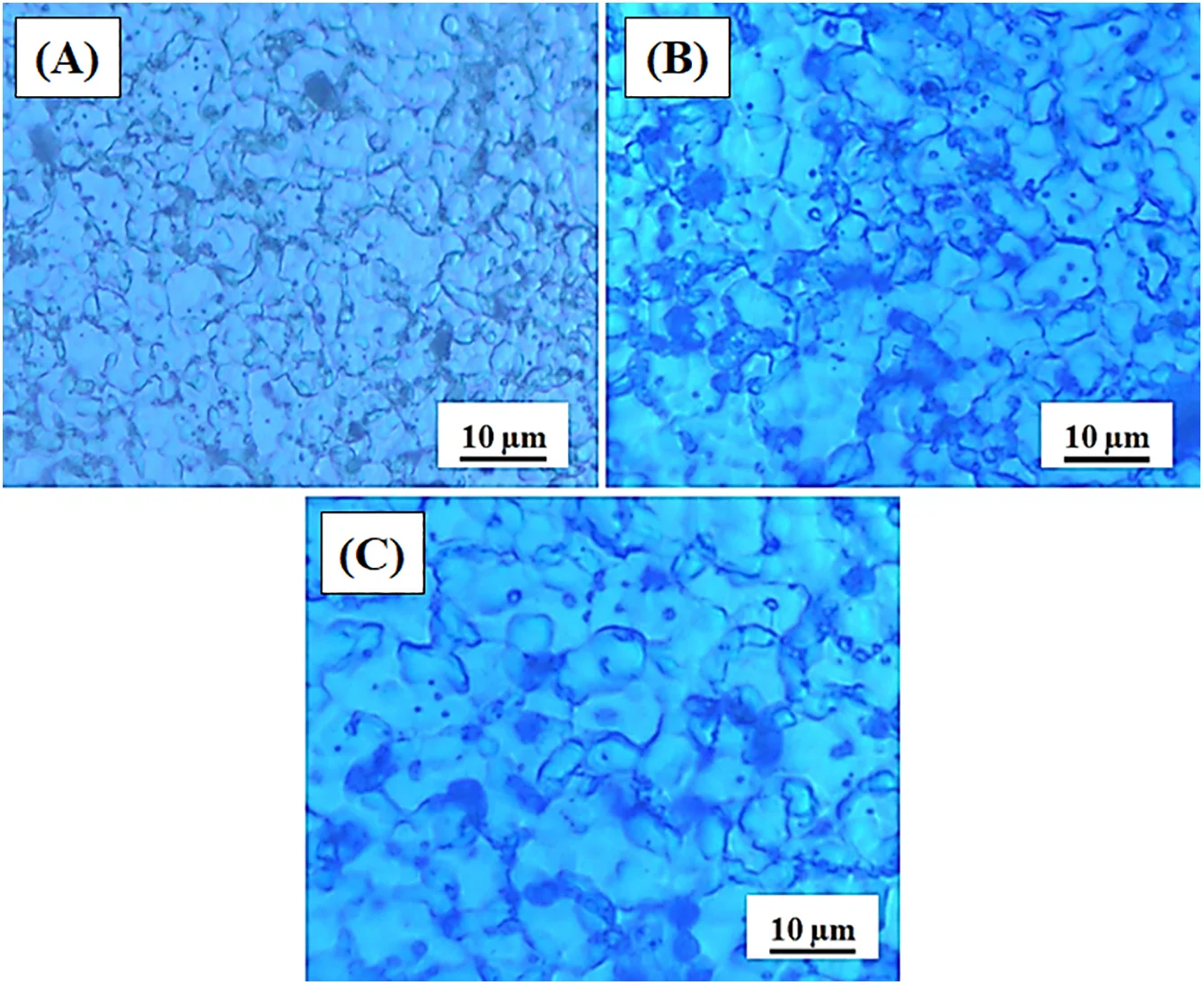
Effect of temperature on grain growth on OM images: (A) ECAP-4P condition, (B) after annealing at 723 K for 6 hours, and (C) after annealing at 723 K for 12 hours.

Moreover, grain growth behavior is evident with prolonged aging at 723 K. The average grain size of the ECAP-4P AZ91 Mg alloy is approximately 7.58 µm, as shown in Fig 6(A). After annealing at 723 K for 6 hours, the grain size increases to 8.06 µm (Fig 6(B)), and further to 8.98 µm after 12 hours (Fig 6(C)) shown in Table 1.

Microhardness and tensile tests were conducted to relate mechanical performance to corrosion resistance. Vickers hardness increased from ~58 HV in the as-received alloy to ~94 HV after ECAP-4P, followed by annealing at 723 K for 12 h. Under the same conditions, tensile strength rose from ~180 MPa to ~265 MPa, accompanied by a slight increase in ductility. These enhancements are attributed to grain refinement, a higher fraction of high-angle grain boundaries, and uniform redistribution of the β-Mg_17_Al_12_ phase. The improved mechanical properties complement the corrosion results, confirming the structural stability of the processed alloys.

### 3.4. Influence of annealing and aging on the corrosion resistance of as-received AZ91 magnesium alloys

Annealing and aging heat treatments were performed on the as-received AZ91 magnesium alloy at temperatures of 523 K, 623 K, and 723 K for durations of 6 and 12 hours. This section compares the microstructure and corrosion properties of heat-treated as-received samples with those of ECAP-processed samples. Additionally, it evaluates the influence of annealing temperature and aging mechanisms on the corrosion behavior of the AZ91 alloy.

**Fig 7:**
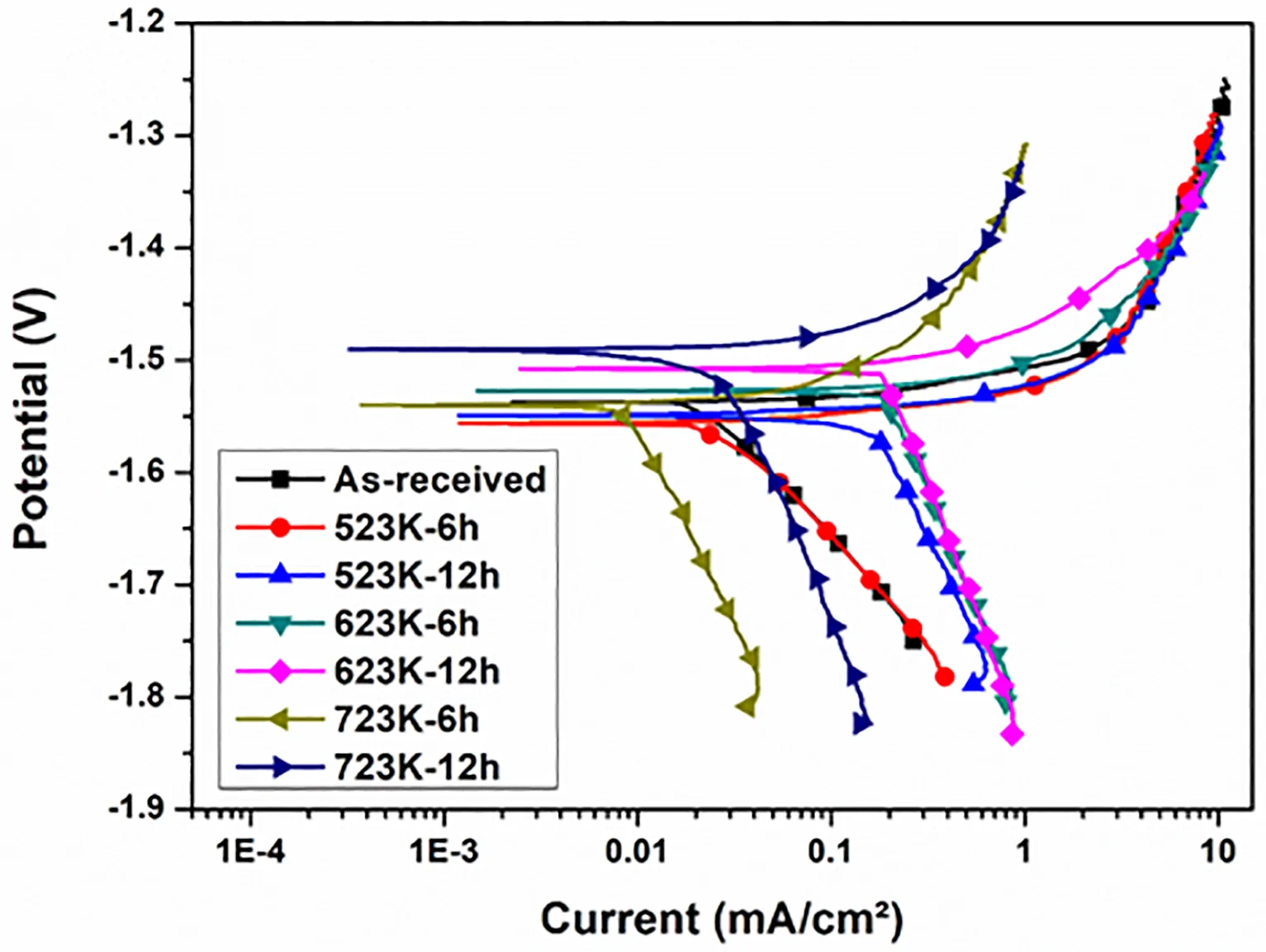
Potentiodynamic polarization plots for heat-treated AZ91 magnesium alloys in a 3.5 wt. % NaCl solution.

Fig 7 shows the potentiodynamic polarization curves for the as-received AZ91 samples and those annealed and aged at the designated temperatures for durations of 6 and 12 hours. The curves indicate a shift toward more positive corrosion potentials for the alloys annealed at 523 K, 623 K, and 723 K, relative to the as-received condition. As shown in Fig 7, the corrosion potential of most heat-treated alloys improved compared to the as-received alloy, except for samples treated at 523 K for both time durations. Notably, the AZ91 alloy annealed at 723 K for 12 hours exhibited a more uniform distribution of secondary phases, resulting in fewer cathodic sites.

As a result, the polarization curve for this sample shifted leftward compared to the as-received and other heat-treated specimens, aligning with findings reported by Liu and Xian-bin et al. [26] for Mg-3Zn alloys. Comparable observations were also made by Taheri et al. [27].

Equation (1) was used to compute the Ecorr, Icorr, and corrosion rate values for the AZ91 alloy samples that were received, annealed, and aged. The results are shown in Table 2. This table consolidates the results from all cathodic polarization tests, as outlined by Zhao et al. [21]. The data indicate that the heat-treated AZ91 alloy exhibited improved corrosion resistance in 3.5 wt.% NaCl solution, which is confirmed through electrochemical results presented in Table 2. This enhancement is attributed to the more uniform secondary phase distribution achieved through annealing and aging, as illustrated in Fig 11.

### 3.5. Corrosion morphologies as-received AZ91 Mg alloys

**Fig 8:**
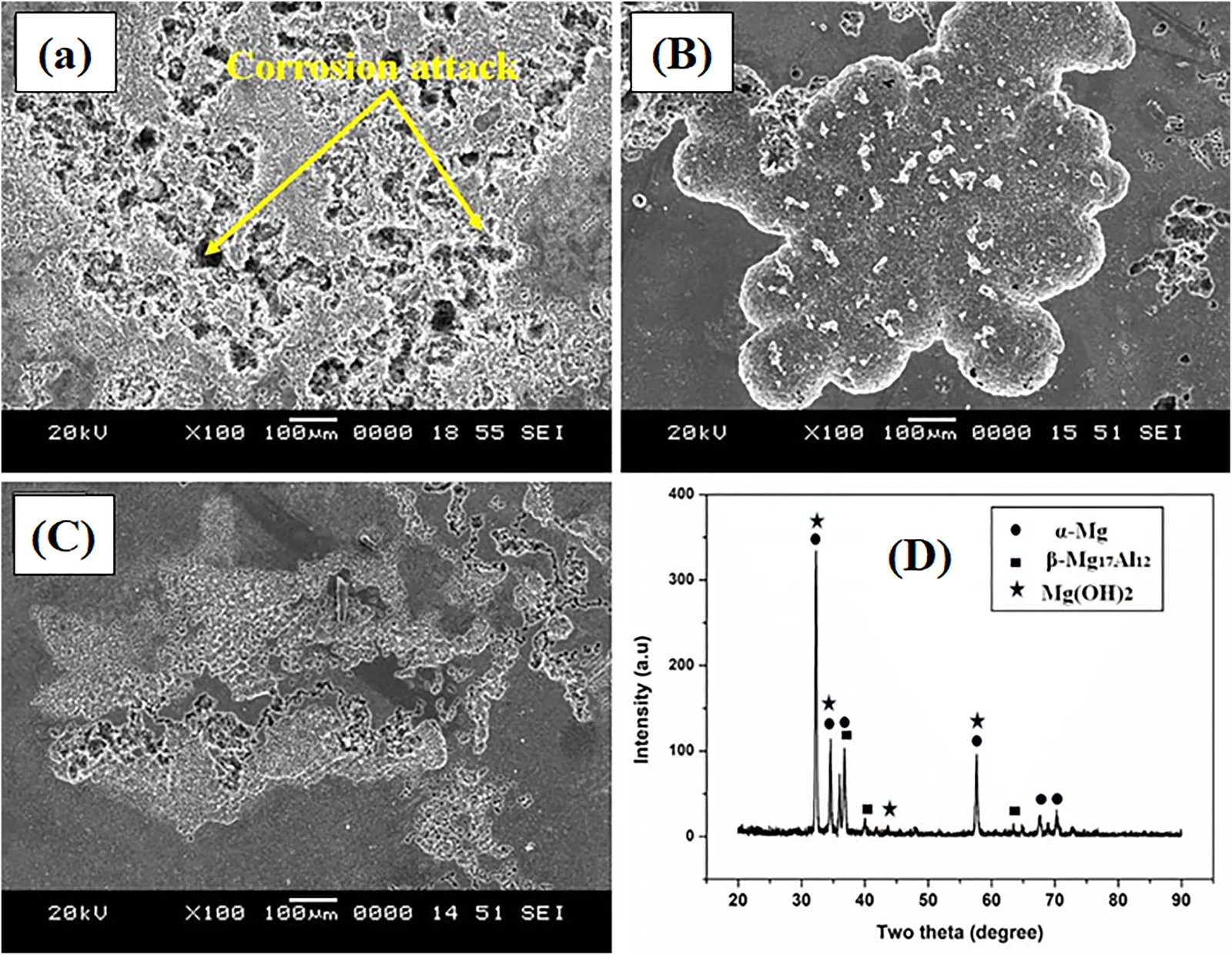
SEM images of corroded AZ91 wrought magnesium alloys: (A) in the as-received state, (B) after annealing at 523 K for 12 hours, (C) after annealing at 723 K for 12 hours, and (D) XRD pattern of the AZ91 alloy annealed at 523 K for 12 hours.

The corrosion surface morphologies of wrought AZ91 magnesium alloys that were received and exposed to 3.5 weight percent NaCl solution are shown in Fig 8, along with specimens that were annealed at 523 K and 723 K for 12 hours. To investigate the extent of corrosion damage, portions of the white corrosion products were carefully removed from the surface of the as-received sample, as shown in Fig 8(A).

The surface morphologies of the samples annealed at 523 K and 723 K for 12 hours, displayed in Fig 8(B) and 8(C), respectively, reveal reduced corrosion damage and uniform corrosion compared to the as-received alloy, along with characteristic pitting corrosion features. Notably, the specimen annealed at 723 K for 12 hours exhibited less surface degradation, attributed to a more uniform distribution of secondary phases achieved during the thermal treatment.

Additionally, Fig 8(D) presents the X-ray diffraction (XRD) pattern of the sample annealed at 523 K for 12 h, confirming the presence of β-Mg_17_Al_12_ phases, with magnesium hydroxide (Mg(OH)_2_) identified as the primary corrosion product. To analyze the degree of corrosion, parts of the white corroded products, mainly consisting of magnesium hydroxide (Mg(OH)_2_), according to the results of XRD analyses shown in Fig 8(D), were extracted from the surface of the as-received specimen, as depicted in Fig 8(A). The observed corrosion features are consistent with the electrochemical polarization data summarized in Table 2.

### 3.6. Effect of annealing and aging processes on the corrosion resistance of ECAP-processed AZ91 magnesium alloys

**Fig 9:**
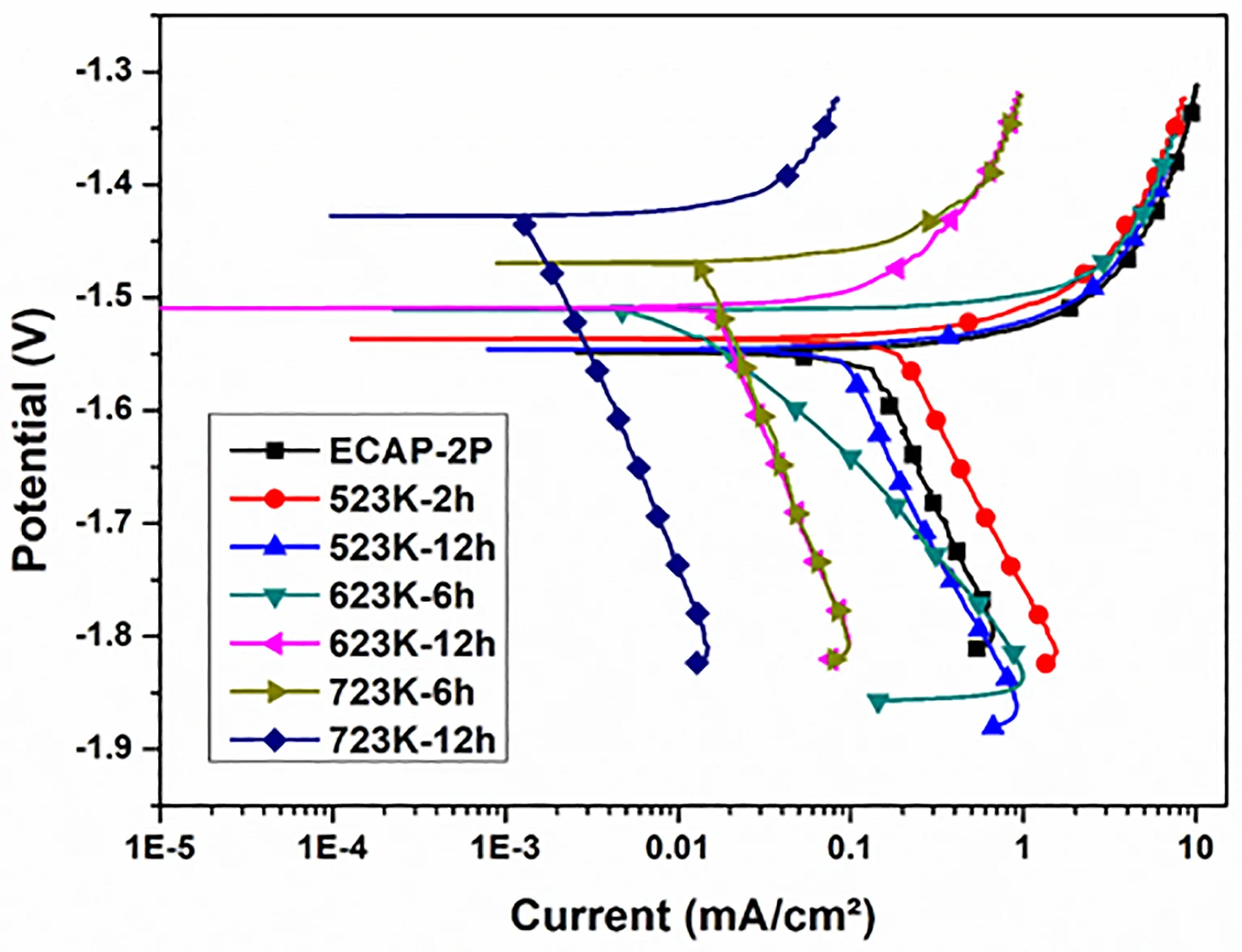
Potentiodynamic polarization curves of heat-treated ECAP-2P AZ91 magnesium alloys tested in a 3.5 wt. % NaCl solution.

**Fig 10:**
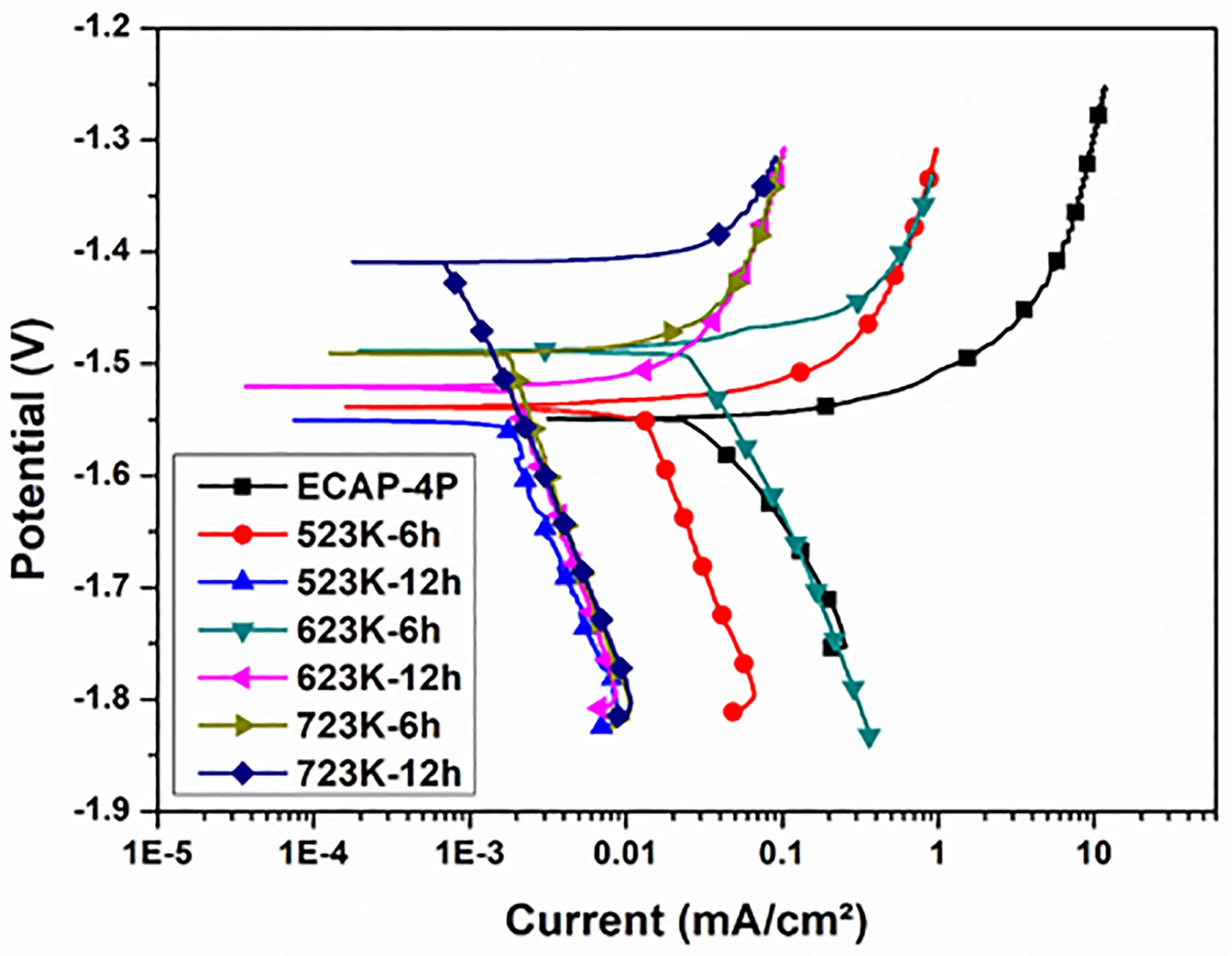
Potentiodynamic polarization profiles of ECAP-4P AZ91 magnesium alloys after heat treatment in a 3.5 wt. % NaCl solution.

Fig 9 and Fig 10 present the polarization curves for AZ91 magnesium alloys processed ECAP-2P and ECAP-4P, both with and without subsequent annealing and aging treatments. While the previous section discussed the corrosion behavior of the as-received alloy subjected to annealing and aging, this section focuses on the effects of these treatments on fine-grained ECAP-processed AZ91 alloys. Comparison of Fig 9 and Fig 10 shows that the polarization curves for heat-treated ECAP-2P and ECAP-4P samples shift noticeably toward more noble potentials, with reduced corrosion current density and elevated corrosion potential, indicating improved corrosion resistance which is confirmed through electrochemical results presented in Table 2.

The Ecorr and Icorr values for both heat-treated and non-heat-treated ECAP-processed AZ91 samples were calculated from the potentiodynamic polarization curves using the Tafel extrapolation method. The corresponding Ecorr, Icorr, and corrosion rate values were determined via Equation (1) and are summarized in Table 2. Fig 9 and Fig 10 present the polarization curves for ECAP-processed AZ91 magnesium alloys that underwent annealing at 523 K, 623 K, and 723 K for durations of both 6 and 12 hours.

The findings show that the lowest values of Icorr (0.00110 mA/cm² and 0.0011 mA/cm²) were recorded for the 723 K-12 h annealed ECAP-2P and ECAP-4P samples, respectively, indicating reduced corrosion susceptibility and a lower rate of corrosion. For all heat-treated samples, the ECAP-4P specimens showed Ecorr values of −1.488 V_SCE_ (room temperature), −1.500 V_SCE_ (6 h at 523 K), −1.512 V_SCE_ (12 h at 523 K), −1.489 V_SCE_ (6 h at 623 K), −1.408 V_SCE_ (12 h at 623 K), −1.402 V_SCE_ (6 h at 723 K), and −1.400 V_SCE_ (12 h at 723 K) are presented in Table 2. These values were lower than those of annealed ECAP-2P Mg alloy, affirming a steady shift towards nobler corrosion potential, which increases corrosion resistance as a result of the annealing treatment. The negative variation of Ecorr for ECAP-4P alloys annealed at 523 K indicates that annealing at lower temperatures initially results in the development of electrochemical heterogeneity within the alloy, owing to partial recovery and redistribution of β-Mg_17_Al_12_ phases. On the other hand, annealing at 623–723 K aids in improving homogeneity, hence better corrosion resistance. Tański et al. observed that annealed alloys showed substantially greater corrosion resistance than bare Al-Mg samples [28]. Naik et al. also discovered that the AZ80 Mg alloy heat-treated at 723 K exhibited greater corrosion resistance compared to the as-received alloy, which is possibly attributed to the uniform distribution of the secondary phases after the annealing treatment [29].

The corrosion current density can be ranked in the order: ECAP-4P > 523 K-6 h > 523 K-12 h > 623 K-6 h > 623 K-12 h > 723 K-6 h > 723 K-12 h. When the ECAP-4P sample was aged for 6 hours at 523 K, the development of Mg_17_Al_12_ phases took place within the α-Mg matrix and on the grain boundaries, resulting in a decrease in the corrosion rate.

**Fig 11:**
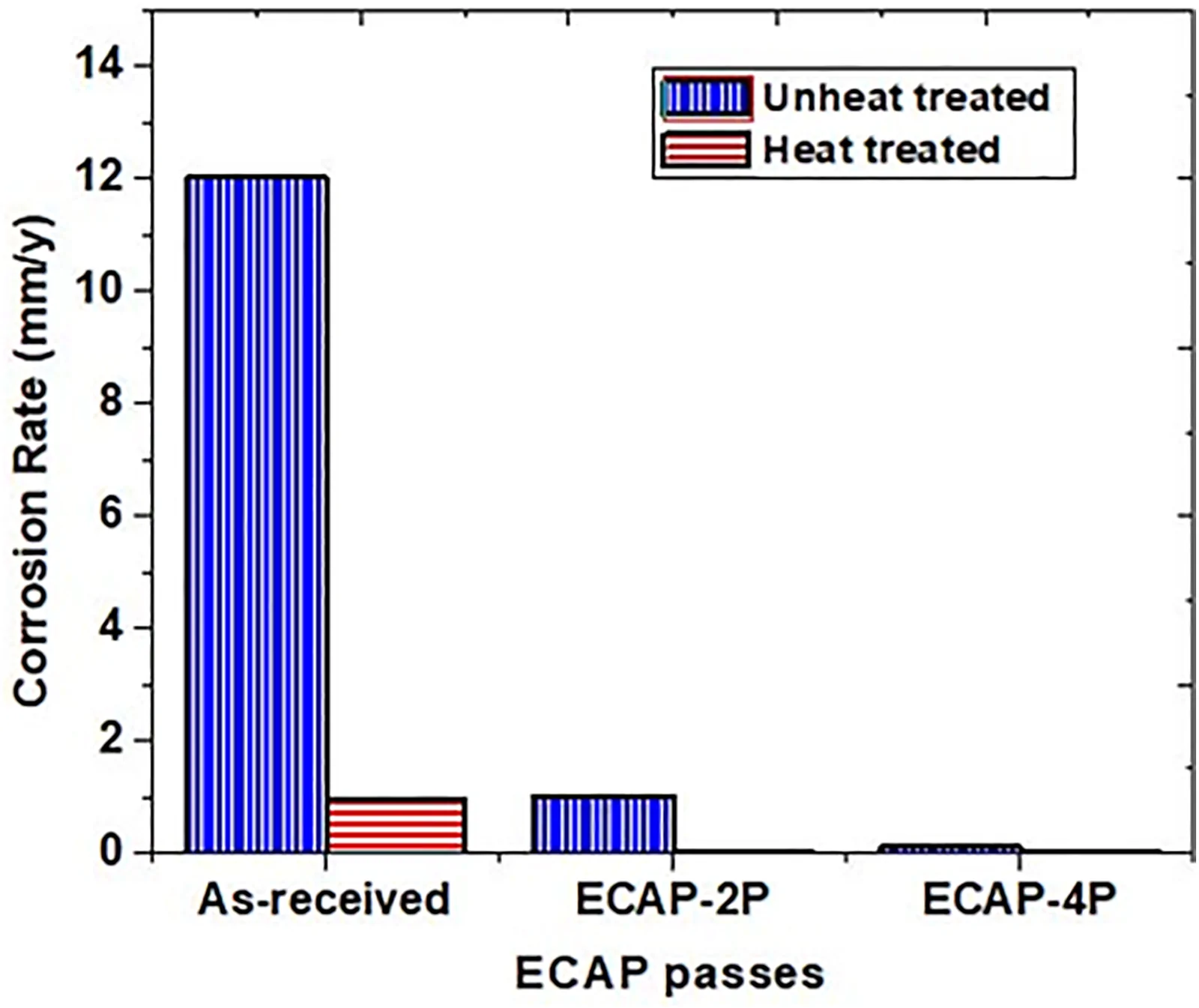
Changes in corrosion rate of AZ91 magnesium alloys before and after annealing at 723 K for 12 hours.

Fig 11 shows the change in corrosion rate for AZ91 Mg alloys before and after heat treatment at 723 K for 12 hours. As aging time was increased from 6 hours to 12 hours, precipitated phases got more evenly distributed, which developed a corrosion barrier and led to the reduction in the corrosion rate. Mg_17_Al_12_ phase distribution was raised after 723 K heat treatment for both 6 and 12 hours, which lowered the number of cathodic sites and thus enhanced corrosion resistance. Similar observations are made by Gopi et al. [30].

### 3.7. Corrosion morphologies of ECAPed AZ91 Mg alloys

**Fig 12:**
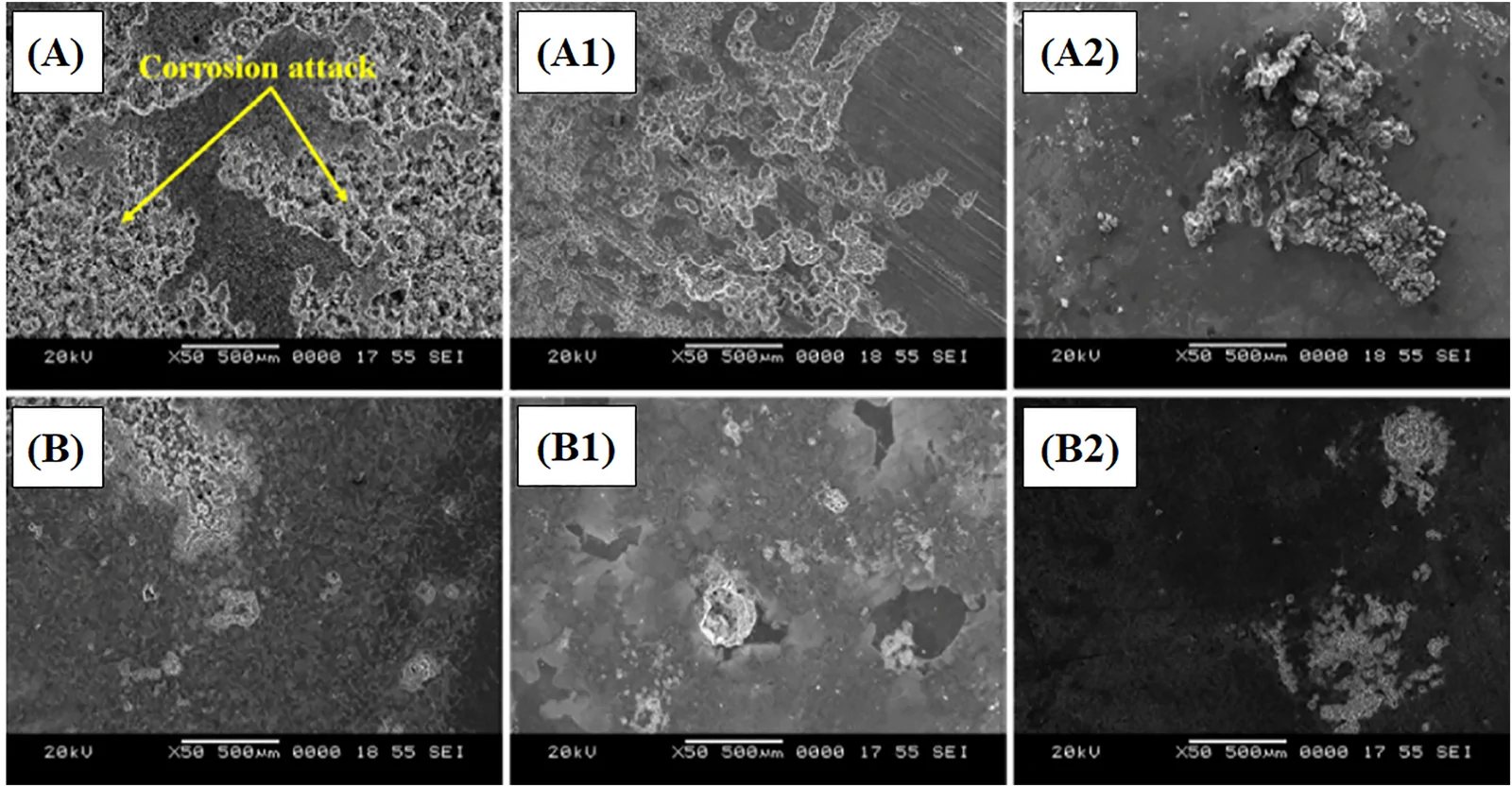
Corrosion surface features of ECAP-processed AZ91 magnesium alloys: (A) ECAP-2P specimens, (A1) after annealing at 523 K for 12 h, (A2) after annealing at 723 K for 12 h; (B) ECAP-4P specimens, (B1) annealed at 523 K for 12 h, (B2) annealed at 723 K for 12 h.

Fig 12 displays SEM images illustrating the corrosion features of AZ91 magnesium alloy samples that underwent ECAP processing and heat treatment, after being exposed to a 3.5 weight percent NaCl solution, as determined by potentiodynamic polarization experiments. The surfaces of heat-treated ECAP samples exhibit significantly reduced corrosion compared to ECAP samples without heat treatment. EDS analysis confirms that the primary corrosion product is Mg(OH)_2_. Corroded surfaces display corrosion pits, clearly visible in Fig 12 (A2). Notably, the ECAP-4P specimen heat-treated for 12 hours at 723 K shows less corrosion damage than the ECAP-2P specimen under the same conditions. Conversely, both ECAP-2P and ECAP-4P samples heat-treated for 12 hours at 523 K, as well as the untreated samples, show more extensive corrosion with deeper pits.

These findings align with the microstructural observations of ECAPed, heat-treated AZ91 alloys, where differences arise from grain refinement and secondary phase distribution. In general, the β-Mg_17_Al_12_ secondary phases exhibit a more noble electrochemical potential compared to the surrounding α-Mg matrix, leading to galvanic corrosion where the secondary phases act as cathodic sites and the Mg matrix as the anode [31,32]. However, after ECAP and subsequent annealing and aging treatments, these secondary phase particles dissolve and redistribute uniformly within the Mg matrix, reducing the number of cathodic sites and minimizing localized corrosion reactions. This promotes uniform corrosion and lowers the overall corrosion rate. Prior studies on the homogenization treatment of the AZ80 Mg alloy as received revealed significant pitting and intergranular corrosion close to the grain boundaries [29]. However, after ECAPed AZ80 Mg alloys were annealed and aged, intergranular corrosion was completely inhibited, leaving only shallow corrosion pits.

The mechanism of corrosion of AZ91 magnesium alloy in a solution of 3.5 wt.% NaCl is mainly controlled by micro-galvanic coupling between the α-Mg matrix and the β-Mg_17_Al_12_ secondary phase. As the electrochemical potential of the β-Mg_17_Al_12_ phase is more noble in comparison to the α-Mg matrix, this phase will act as a cathode, while the α-Mg matrix will act as an anode, which will result in the preferential dissolution of Mg. Chloride ions will penetrate and break down the Mg(OH)_2_ film, which is responsible for the corrosion of magnesium [33]. In the as-received alloy, the continuous β-phase at grain boundaries will intensify galvanic attack and intergranular corrosion. However, after the alloy undergoes the treatment of Equal Channel Angular Pressing (ECAP) and subsequent annealing, the grain structure is refined and the β-Mg_17_Al_12_ phase is homogeneously distributed, which will reduce the intensity of cathodic sites and localized galvanic interactions, resulting in a more uniform corrosion rate.

Previous studies on AZ31 magnesium alloy have shown that subsequent annealing treatments have a significant influence on the electrochemical behavior of materials processed via extrusion and ECAP. In this study, potentiodynamic polarization tests of AZ31 magnesium alloy processed via ECAP and annealed at various conditions for 1 hour in the range of 150 °C to 250 °C in 0.1 M NaCl solution showed an increase in polarization resistance. The results showed an increase of approximately 17% in polarization resistance for the specimen annealed at 250 °C in comparison to the ECAP-processed condition. Microstructural observations using SEM and EBSD showed an improvement in microstructural stabilization, whereas positron annihilation spectroscopy results showed a gradual decrease in dislocation density. The improvement in the electrochemical behavior of magnesium alloys was attributed to the combined effects of reduced lattice defects, dissolution of second-phase particles, and micro-galvanic effects [34].

Although the current research has shown clear correlations between ECAP, thermal treatment and the corrosion properties of the AZ91 magnesium alloy, it is necessary to note the limitations of the current research. Firstly, the crystallographic texture of the alloy was not investigated by means of electron backscattering diffraction, which could have provided more information about the influence of texture on the anisotropy of the corrosion properties of the alloy. Secondly, the corrosion properties of the alloy were investigated by means of potentiodynamic polarization curves, while EIS and immersion tests were not carried out to validate the protective properties of the alloy under long-term exposure. Thirdly, the microstructural investigations have provided information about the distribution of the β-Mg_17_Al_12_ phase, while the quantitative content of the β-Mg_17_Al_12_ phase was not investigated.

## 4. Conclusions

The study demonstrates that the synergistic effects of ECAP and heat treatments enhance the corrosion resistance of AZ91 magnesium alloy through microstructural refinement and uniform phase distribution. These findings provide insight into optimizing processing parameters to achieve improved performance in corrosive environments, enabling broader applications of AZ91 Mg alloy where weight reduction and corrosion resistance are critical. The key findings are summarized below:

- ECAP processing significantly refined the grain structure, reducing the grain size from 58.69 µm to 7.58 µm (ECAP-4P), which improved the structural stability of the AZ91 alloy.
- ECAP combined with annealing produced a more uniform distribution of β-Mg_17_Al_12_ phases, increasing their fraction by ~30% at 723 K for 12 h, thereby reducing localized galvanic corrosion.
- Corrosion potential (Ecorr) improved from −1.539 V to −1.400 V, showing a ~ 9% positive shift, which indicates enhanced electrochemical stability after thermal treatment.
- Corrosion current density (Icorr) decreased drastically from 0.2630 to 0.0011 mA/cm², demonstrating a ~ 99.58% improvement in corrosion resistance for the ECAP-4P + 723 K/12 h condition.
- SEM corrosion analysis showed about 75% reduction in pitting, confirming that the combined ECAP and annealing treatment effectively enhances corrosion resistance of AZ91 Mg alloy.

These results provide strong evidence supporting the use of ECAP and tailored heat treatment strategies to improve corrosion resistance and extend the applicability of AZ91 magnesium alloys in demanding service environments. The improved properties of corrosion resistance, which are imparted by the ECAP and thermal treatment processes, have made the AZ91 magnesium alloy an important material for application in automobile, aerospace and lightweight structures, especially where there is a need for resistance to corrosion and low weight.
