# Supplementary material for: Synergistic impact of ECAP and thermal treatments on the corrosion performance of AZ91 Mg alloy
Source: PLoS One. 2026 Sep 25;21(9):e0354359. doi: 10.1371/journal.pone.0354359 (PMC13614571; doi:10.1371/journal.pone.0354359)
Supplement: Supplementary file 2 [file pone.0354359.s003.pdf]

Review on “Synergistic Impact of ECAP and Thermal Treatments on the Corrosion Performance of AZ91 Mg Alloy”

The authors have attempted to systematically investigate the mechanical and corrosion behavior of AZ91 Mg alloy by performing ECAP 2P, 4P and heat treatment at various temperatures and time. While the work is interesting for PLOS One, there is a lot of scientific issues that are unclear and needs clarification before consideration for publication. **The referee recommends the work to be considered for publication after major revision**.

Here are some of the points the authors must address for future consideration:

1. Please guide the readers by clearly marking the secondary phases, eutectic phases, residual phases or any other phases in a microstructure before describing the details.

2. In many instances, the authors have mentioned that the corrosion resistance is improved in Section 3 without any supporting data. Please conclude only if you have supporting evidence or wait till the evidence is shown to conclude on corrosion resistance.

3. Figure 1 shows the SEM image of the as-received, 2P and 4P microstructures. While the as-received microstructure is shown at x1000, the other two are shown at a higher magnification. It is generally better to show the same magnification is available. While the scale bar should be used to analyze the images independently, since these are shown side-by-side, a quick glance may indicate that the grain sizes are the similar for all cases. If the authors want to keep the same images, please include an average ASTM grain size calculated for all the three cases both in the Figure caption and in the text for comparison. This will support the conclusion in the last Paragraph of Page 5 on grain refinement due to 2P and 4P with quantitative numbers.

4. In Figure 1(a), the claim of coarse-grained structure is accepted, but it is better to quantify it with the comment in 1. In addition, the claim that there are high fraction of secondary phases along the grain boundaries are not clear at all. Please highlight the areas or provide supporting evidence of this grain boundary segregation.

5. In Page 5, last paragraph, the authors have reported that ECAP generates high density of dislocations and activates dynamic recrystallization. There is no supporting data shown for this conclusion. Please include the images showing dislocations or include references to support this claim.

6. The conclusions reported in the last Paragraph of Section 3.1 (Page 6) is very confusing. It is unclear how one can assign shear planes, basal texture, etc. Please clarify.

7. Page 6, Section 3.2, 1st Paragraph: It is unclear how to compare the secondary phases in all the heat treated microstructures. Looking at the as-received one in Figure 1(a) with respect to the ones in Figure 2, please highlight which is a secondary phase, how is the

eutectic structure identified in these images? What is the eutectic structure is also not mentioned appropriately. Please guide the reader so that it is easy to understand the impact of heat treatment in these alloys.

8. From Figure 2, it is also unclear on how much refinement is done with different temperatures mentioned in the text. It is also unclear how the continuity of the secondary phases are reported here by looking at a 2D image.

9. There is some grain size measurement reported in Section 3.2 with heat treatment. However, the numbers reported does not seem to match at least the as-received sample microstructure shown in Figure 1(a). It looks like the grain size of Figure 1(a) should be between 20 - 35 µm. How did the authors get a 58.69 µm for the as-received sample in Figure 3(a). Please keep the data consistent over the various portion of the discussion. Kindly clarify.

10. In Page 10, 1st Paragraph, the authors have mentioned reduction of residual phases. What are these? Kindly define every possible phase that are discussed in a better way so that the audience can keep track of all the changes in the microstructure based on the supporting evidence.

11. Page 10, 2nd Paragraph also refers to the increased fractions of the β-phase without any clear guidance from the SEM images shown.

12. Why Figure 6 has a blue filter? Please include SEM in the caption.

13. The authors have reported the Mechanical properties in the last paragraph of Section 3.3. It will be better to include the stress-strain diagram for these and mention the sample preparation for these tests. Also, for hardness, it is necessary to include all the different cases reported so far and not just as-received and one of the heat treated 4P samples.

14. Figures 7, 9, and 10 needs to be improved. It is not very easy to see the trends based on the way the data is plotted (with the symbols and lines combinations used for the plots).

15. In Page 14, the authors have referred to Equation (1) to compute E_{corr}, I_{corr}, and Corrosion Rate. However, Equation (1) is not reported in the text. This type of overlook is not appropriate. Please correct.

Kindly update the recommended modifications and go through the corrosion data while finalizing the article for re-evaluation. Here are some minor corrections to implement:

1. Page 2, Introduction, 1st Paragraph, Line 10: "…., contributing to enhanced strength and ductility". Please make sure both strength and ductility were enhanced at the same time or if strength is enhanced ductility is decreased.

2. Page 8, Introduction, 2nd Paragraph: starts with "Recent investigations indicate that ECAP….". This is supported by Reference [11], which is from 2021. This is not recent. Also, "investigations" mean there has to be more than one article referred for that statement. Please update.
3. Page 3, 2nd Paragraph, Line 7: Please rewrite "…significant international progress…". It is unclear what this means.
4. Page 4, Last Paragraph: Define "OM" before using. Is it optical microscope?
5. In Page 4, the authors have reported ECAP was performed at 325 °C, while in Page 5 Section 3.1, 1st Paragraph the authors have indicated the ECAP done at 598 K. This is confusing. Please use the same temperature unit so that one do not need to convert from one unit to the other.
