# Supplementary material for: Synergistic impact of ECAP and thermal treatments on the corrosion performance of AZ91 Mg alloy
Source: PLoS One. 2026 Sep 25;21(9):e0354359. doi: 10.1371/journal.pone.0354359 (PMC13614571; doi:10.1371/journal.pone.0354359)
Supplement: Supplementary file 4 [file pone.0354359.s006.pdf]

Review on the revised version of "Synergistic Impact of ECAP and Thermal Treatments on the Corrosion Performance of AZ91 Mg Alloy"

The authors have updated the content to improve the article based on the recommendation of various referees. While the work is interesting for PLOS One, there is still scope to improve and clarify details before publication. **The referee recommends the work to be considered for publication after the following minor revision**.

Here are some of the points the authors should address for future version:

1. Please provide the quantitative values for grain sizes related to Figure 1 for better understanding in Section 3.1.
2. Figure 2 still contains a lot of information which is unclear. It will be great if the authors could add a supplementary section and provide high resolution individual images where the different aspects highlighted in the images are completely clear. The authors have labeled α-Mg, Petal Shape, Eutectic Phase, Discontinuous distribution of secondary phase. This is true for any other images shown later where the authors have labeled different phases or features to be added in the supplementary section for clearer reference.
3. It is unclear what type of imaging is used for Figure 3 (Optical microscopy is mentioned in part of the text). Please add it in the caption and in the text. Also, include the grain sizes in the figure caption.
4. Please refer to Tables 1 and 2 wherever necessary in the results section to guide the readers on the original contribution better. For example, in the discussion of Figure 3, the authors can refer to Table 2 for grain size measurement.
5. Page 9, Section 3.3, 1st Paragraph, Line 4: sentence referring to Figure 4 for hear-treated as-received alloy. Figure 4 does not have any as-received alloy micrographs. Please update as needed.
6. Similar to the previous version, the authors are consistently indicating some phases are better corrosion resistant without the corrosion data. It feels like, it would be better to first show the corrosion data and then present the microscopies so that the text will have more meaning. For example, in Section 3.3, Page 10, 1st Paragraph, last sentence clearly concludes corrosion related performance by only referring to the micrographs, which is confusing.
7. In Page 10, Section 3.5, 1st Paragraph, the authors mentioned "the white corrosion products". Is there a chemical composition that can be referred here?
8. Based on the images shown in Figure 8, the as-received sample shows more corrosion than the annealed samples, is that right? If this is correct, then in the text Page 15, Section 3.5, 2nd Paragraph, first sentence indicates that Figures 8b) and c) reveal more extensive corrosion compared to the as-received alloy needs to be updated.
9. Please replot Figures 7, 9 and 10 to make it clearer. One way to do it is to keep the symbols the same for the same temperature and use a filled symbol for 6 h and open symbol for 12 h or vice versa. Also. the lines used to join the data points sometimes look unnatural. For example, the 623 K – 6 h data shown in Figure 9

10. Page 17, 2nd Paragraph, Line 1: the authors mentioned "….lowest values of Icorr (0.0110 mA/cm^2 and 0.0011 mA/cm^2) were….". One of these current values do not match the data in the Table. Please correct.
11. Please move Figure 11 to the supplementary information as it is redundant. The data is well presented in Table 1.
12. Page 21, the paragraph starting with "Although the current research…." Is this talking about the work of the current article? Please clarify. This complete paragraph looks disconnected.
13. What is the error bar of the data presented in Table 2? Please give a range or standard deviation to understand the spread in grain sizes.
14. There are some interesting patterns in Table 1 that the authors should indicate or discuss. For example, why E_{corr} of ECAP-4P gets worse from Room temperature to 523 K 6 h and 523 K 12 h, before getting better for 623 K onwards.

Here are some minor corrections to implement:

1. Page 2, Introduction, 2nd Paragraph, Line 1: Recent investigations indicate….". Please refer some of the recent articles for supporting this sentence.
2. Page 3, Introduction, 2nd Paragraph, Line 5: "Recent efforts in the field….". It referes to an article from 2021 [Ref 11]. This is not regarded as recent.
3. Page 3, Last Paragraph. Line 3: Replace "severe plastic deformation" with SPD.
4. Page 4, Section 2, 1st Paragraph: font size of last few lines are smaller than the rest.
5. Page 5, Line 3: EDS is used for the first time. Please use the full form here rather than later (done in Page 5, Line before Equation 1)
6. Page 7, Section 3.2, 1st Paragraph, Line 5: Fix the formatting of β-Mg17Al12
7. Page 10, 1st Paragraph has most of the information in the beginning covered in the first two paragraphs of Section 3.3 in Page 9. Please remove any redundancy.
8. Page 10, Last Line: Rewrite "shown in Table 2"
9. Page 16, Last Paragraph: "two passes (ECAP-2P) and four passes (ECAP-4P)" Please use the acronym once mentioned earlier.
10. Page 20, 2nd Paragraph: Fix the formatting of β-Mg17Al12 and Mg(OH)2.
11. Page 20: Please use ECAP, which is well established before rather than Equal Channel Angular Pressing
12. Page 20, Last Paragraph, 1st Sentence needs reference. Without it, the second sentence "ln this study, potentiodynamic…." becomes confusing. It feels like it is the work of the current authors.
13. Please check the header formatting of Table 1, say Annealing Time (h) is wrapped in a confusing way.
