# Supplementary material for: Synergistic impact of ECAP and thermal treatments on the corrosion performance of AZ91 Mg alloy
Source: PLoS One. 2026 Sep 25;21(9):e0354359. doi: 10.1371/journal.pone.0354359 (PMC13614571; doi:10.1371/journal.pone.0354359)
Supplement: Supplementary file 5 [file pone.0354359.s002.pdf]

Review on manuscript PONE-D-25-66592

of paper "*Synergistic Impact of ECAP and Thermal Treatments on the Corrosion Performance of AZ91 Mg Alloy*"

The article explore the corrosion behavior of AZ91 magnesium alloy subjected to heat treatments after effects of equal-channel angular pressing; and it is in accordance with journal topics.

The title reflects the content of the article, and the abstract provides a brief summary of the research purpose. This study examines the synergistic effects of equal-channel angular pressing and heat treatments on the corrosion behavior of AZ91 magnesium alloy. The alloy was subjected to effects of equal-channel angular pressing for two and four passes to refine the microstructure, followed by annealing and aging treatments at three different temperatures (523 K, 623 K, and 723 K) for durations of 6 and 12 hours. Microstructural characterization, including grain structure and secondary phase distribution, was conducted using optical microscopy, scanning electron microscopy, and energy-dispersive X-ray spectroscopy. Corrosion behavior was evaluated using potentiodynamic polarization tests in a 3.5 wt.% NaCl solution.

However, some statements are unclear and the manuscript needs revision and improvement.

Remark 1:
The introduction section requires significant improvement. It should include a comprehensive and thorough review of the current state of the art in the field of study. Incorporating a SWOT analysis is highly recommended. Additionally, please introduce and discuss more recent works. In the final paragraph of this section, emphasize the novelty and scientific significance of this study in advancing the field.

Remark 2:
The keywords should clearly reflect the primary materials, tests, and methodologies utilized in the study. It is necessary to revise the keywords accordingly, ensuring they align with the criteria mentioned above.

Remark 3:
It is recommended to avoid acronyms in title, keywords and abstract section of the paper. The acronyms must be defined where appear for the first time in the text beginning from introduction section.!!!!!!
For *energy dispersive X-ray spectroscopy* the authors used EDX and EDS acronym!!!

Remark 4:
The manuscript presents results from several experimental techniques (SEM, OM, EDS, corrosion, tensile, and hardness tests), but lacks information regarding the instruments employed. The authors are encouraged to specify the equipment in the Materials and Experimental Procedure section!!!!!!

Remark 5:
Certain conclusions appear ambiguous and lacking in substance. They are overly general and warrant revision and enhancement. Presently, they merely serve as summaries. It is imperative that the conclusion section emphasizes the scientific contributions made by the article and the practical relevance of the findings and results.

In conclusion, I consider that the article have too many errors which need to be corrected and present a medium level of scientific value/novelty. Due to remarks presented above, I cannot recommend to publish the manuscript in this form. Please revise.
