# Supplementary material for: Synergistic impact of ECAP and thermal treatments on the corrosion performance of AZ91 Mg alloy
Source: PLoS One. 2026 Sep 25;21(9):e0354359. doi: 10.1371/journal.pone.0354359 (PMC13614571; doi:10.1371/journal.pone.0354359)
Supplement: Supplementary file 7 [file pone.0354359.s007.pdf]

The manuscript presents a relevant topic and contains potentially useful findings. However, in its current form, it requires **major revision** before it can be considered for publication. The primary concerns include an insufficient demonstration of novelty, limited depth in corrosion analysis, a lack of supporting advanced electrochemical characterization, and a largely qualitative rather than quantitative microstructural interpretation. Several conclusions are presented without adequate statistical or mechanistic support. Additional validation methods, more apparent justification of processing parameters, improved image quality, and a deeper mechanistic explanation are required to substantiate the claims. With substantial strengthening of methodology, data interpretation, and presentation clarity, the manuscript may reach publishable quality, but significant development is still needed at this stage.

- The study is interesting, but the novelty could be articulated more clearly to distinguish it from existing works on AZ91 corrosion improvement.

  Response: The novelty lies in the systematic integration of ECAP (2P and 4P) with controlled annealing treatments (523–723 K) to establish a direct quantitative correlation between grain refinement, β-$Mg_{17}Al_{12}$ redistribution, and corrosion performance in AZ91 alloy. Unlike prior studies that examined ECAP or heat treatment independently, this work demonstrates the synergistic effect of combined processing, achieving ~99% reduction in corrosion rate and providing structure–corrosion relationships under identical processing conditions. Mentioned in introduction section

- The manuscript would benefit from a more substantial justification of why the specific ECAP passes and heat-treatment conditions were selected.

  Response: The Introduction has been revised to explicitly differentiate this work from prior studies by highlighting the combined ECAP + controlled annealing approach and its quantitative corrosion improvement.

  The heat-treatment temperatures (523–723 K) were chosen based on the precipitation and stability range of the β-$Mg_{17}Al_{12}$ phase. Specifically, 523 K promotes initial precipitation, 623 K enables redistribution and coarsening, and 723 K approaches homogenization without reaching the incipient melting temperature.

- The corrosion analysis is useful but could be strengthened by incorporating additional techniques, such as EIS or immersion testing, to support the polarization results.

  Response: Potentiodynamic polarization was selected to quantitatively determine Ecorr, Icorr, and corrosion rate, enabling direct comparison between processing conditions. While techniques such as EIS or long-term immersion testing could provide additional insight into film stability and degradation kinetics, the polarization results are internally consistent and supported by SEM corrosion morphology analysis.The absence of EIS/immersion testing is acknowledged as a limitation and suggested for future work.

- Providing statistical validation or multiple repeat trials would enhance confidence in the reported data.

  Response: All electrochemical tests were performed in triplicate under identical conditions, and the reported values represent averaged results. The experimental deviation was within acceptable limits for corrosion testing of magnesium alloys. This clarification has been added to the methodology section to strengthen statistical reliability.

- The discussion on the synergistic effect is promising but would benefit from clearer evidence or a quantitative demonstration.

  Response: The synergistic effect is quantitatively demonstrated through the combined reduction in grain size (58.69 µm → 7.58 µm) and corrosion current density (0.2630 → 0.0011 mA/cm^2), resulting in ~99% reduction in corrosion rate. This simultaneous microstructural refinement and β-$Mg_{17}Al_{12}$ redistribution confirms that corrosion improvement arises from the integrated ECAP–annealing treatment rather than individual processing steps.

- Some claims appear stronger than the supporting data; tempering the language would improve scientific balance.

  Response: The manuscript has been revised to moderate strong or generalized statements. Conclusions are now strictly aligned with experimentally measured data (Ecorr, Icorr, grain size, hardness).

- The microstructural analysis could be improved with more quantitative metrics, such as grain size distribution or phase fraction estimation.

Response: Quantitative grain size measurements were performed using the ASTM E112 line intercept method and average grain size values are now reported for all key conditions.

Additionally, relative β-$Mg_{17}Al_{12}$ phase fraction trends were discussed based on comparative SEM analysis. The manuscript has been revised to incorporate these quantitative metrics to strengthen microstructural interpretation.

- Including EBSD or additional crystallographic information may help better explain the recrystallization and texture evolution.

Response: While EBSD would provide detailed crystallographic texture and recrystallization analysis, the present study focused primarily on microstructure–corrosion correlation. Grain refinement and phase redistribution were validated using OM and SEM observations.

The absence of EBSD characterization is acknowledged as a limitation and detailed texture analysis is proposed as part of future work.

- SEM images are informative, but slightly higher resolution images or clearer contrast may improve visual interpretation.

Response: Higher-resolution SEM images with improved contrast have been included in the revised manuscript. Image annotations and scale bars were also enhanced to improve phase visibility and overall microstructural interpretation.

- The corrosion mechanism explanation could be expanded to provide deeper electrochemical insight and stronger scientific linkage.

Response: The corrosion mechanism discussion has been expanded to clarify the galvanic interaction between the α-Mg matrix (anodic) and β-$Mg_{17}Al_{12}$ phase (cathodic). The role of grain refinement in promoting uniform corrosion, reducing localized galvanic coupling, and stabilizing the surface $Mg(OH)_2$ film has been explained to provide stronger electrochemical linkage with the polarization data.

- The relationship between β-phase redistribution and corrosion performance would benefit from a more detailed mechanistic explanation.

Response: The manuscript now clarifies that in the as-received condition, β-$Mg_{17}Al_{12}$ forms a continuous network along grain boundaries, acting as strong cathodic sites and promoting localized galvanic corrosion. After ECAP and annealing, the β-phase becomes finer and more uniformly dispersed within the α-Mg matrix, reducing cathodic intensity, interrupting galvanic continuity and promoting more homogeneous corrosion. This redistribution lowers localized pit initiation and decreases overall corrosion current density, directly linking phase morphology to improved corrosion performance.

- More detailed comparison with related recent studies would help highlight where this work stands in the broader research landscape.

  Response: A concise comparison has been added summarizing corrosion rates, processing methods (ECAP/annealing/other SPD), and microstructure–property relationships from recent studies on similar Mg alloys. This comparison highlights how the current integrated ECAP + controlled annealing approach achieves superior corrosion reduction and provides clearer structure–performance linkage compared to individual processing strategies reported in the literature.

- A clearer separation between observation and interpretation in the results section would improve readability.

  Response: The Results section has been reorganized to clearly distinguish experimental observations (micrographs, measured values, polarization data) from mechanistic interpretation. Descriptive findings are now presented first, followed by a separate analytical discussion to improve clarity and scientific readability.

- Adding a brief limitations section could help provide perspective and transparency to the study.

  Response: A dedicated limitations section has been incorporated, noting the absence of EBSD-based texture analysis, EIS/long-term immersion validation, and precise quantitative phase fraction measurements. These factors are recognized as scope boundaries of the present work and are proposed as future extensions to further reinforce the microstructure–corrosion relationship.

- Figures are useful, but more consistent annotation formatting would enhance presentation quality.

Response: All figures have been reformatted to ensure consistent font size, labeling style, arrow markings and scale bar presentation. Captions were standardized and phase annotations were uniformly applied to improve clarity and overall presentation quality.

- The narrative flow could be slightly tightened in places to make the discussion more concise and impactful.

  Response: The manuscript has been carefully edited to remove redundant statements and streamline transitions between sections. The discussion has been tightened to focus on key quantitative findings and mechanistic insights, improving clarity and overall impact.

- Including a discussion of long-term stability or environmental relevance would increase the practical value of the findings.

  Response: A brief discussion on long-term corrosion stability and environmental relevance has been added. The potential applicability of the optimized ECAP + annealing condition for chloride-rich environments such as marine, aerosace and automotive exposure is highlighted, while noting that extended immersion studies are recommended for future validation.

- Some sections could benefit from clearer transitions to guide the reader logically through the results.

  Response: Transitional sentences have been added between microstructural characterization, mechanical properties and corrosion analysis sections to improve logical continuity. The revised structure now guides the reader sequentially from processing, microstructure evolution and corrosion performance.

- The conclusion effectively summarizes the findings but could be more explicitly linked to industrial or application relevance.

  Response: The conclusion has been revised to explicitly link the improved corrosion resistance and mechanical strengthening to lightweight structural applications in automotive, aerospace, and marine environments. The practical significance of the optimized ECAP + annealing condition is now clearly stated in terms of enhanced durability and service life in chloride-containing conditions.

- Overall, the work shows promise and contains meaningful observations. With some strengthening of analysis and presentation, it has good potential.

  Response: We appreciate the positive assessment. The manuscript has been substantially strengthened through improved quantitative analysis, clearer mechanistic explanation, enhanced figure quality and moderated conclusions.

Review on "Synergistic Impact of ECAP and Thermal Treatments on the Corrosion Performance of AZ91 Mg Alloy"

The authors have attempted to systematically investigate the mechanical and corrosion behavior of AZ91 Mg alloy by performing ECAP 2P, 4P and heat treatment at various temperatures and time. While the work is interesting for PLOS One, there is a lot of scientific issues that are unclear and needs clarification before consideration for publication. The referee recommends the work to be considered for publication after major revision. Here are some of the points the authors must address for future consideration:

1. Please guide the readers by clearly marking the secondary phases, eutectic phases, residual phases or any other phases in a microstructure before describing the details.

Response: All microstructures have been revised with clear annotations identifying the α-Mg matrix, β-$Mg_{17}Al_{12}$ secondary phase, eutectic (α + β) regions, and residual particles. Arrows and labeled insets are added in the figures and phase descriptions are clarified in the captions prior to detailed discussion in the text.

2. In many instances, the authors have mentioned that the corrosion resistance is improved in Section 3 without any supporting data. Please conclude only if you have supporting evidence or wait till the evidence is shown to conclude on corrosion resistance.

Response: The manuscript has been revised to ensure that all statements regarding corrosion resistance are made only after presenting supporting electrochemical data (Ecorr, Icorr, and corrosion rate).Premature conclusions have been removed and interpretations are now strictly aligned with the corresponding experimental evidence.

3. Figure 1 shows the SEM image of the as-received, 2P and 4P microstructures. While the as-received microstructure is shown at x1000, the other two are shown at a higher magnification. It is generally better to show the same magnification is available. While the scale bar should be used to analyze the images independently, since these are shown side-by-side, a quick glance may indicate that the grain sizes are the similar for all cases. If the authors want to keep the same images, please include an average ASTM grain size calculated for all the three cases

both in the Figure caption and in the text for comparison. This will support the conclusion in the last Paragraph of Page 5 on grain refinement due to 2P and 4P with quantitative numbers.

Response: Figure 1 has been revised to ensure consistent magnification for direct comparison. Additionally, average grain size values measured using the ASTM E112 line intercept method are now included in both the figure caption and text (as-received: 58.69 µm; 2P: 30.86 µm; 4P: 7.58 µm). This quantitative comparison clearly supports the conclusion on progressive grain refinement after 2P and 4P ECAP processing

4. In Figure 1(a), the claim of coarse-grained structure is accepted, but it is better to quantify it with the comment in 1. In addition, the claim that there are high fraction of secondary phases along the grain boundaries are not clear at all. Please highlight the areas or provide supporting evidence of this grain boundary segregation.

Response: The coarse-grained structure in Figure 1(a) is now quantitatively supported with the measured average grain size (58.69 µm) using the ASTM E112 method.Grain boundary β-$Mg_{17}Al_{12}$ segregation has been highlighted with annotated regions in the SEM image, and compositional confirmation from EDS analysis is referenced to support phase identification.

5. In Page 5, last paragraph, the authors have reported that ECAP generates high density of dislocations and activates dynamic recrystallization. There is no supporting data shown for this conclusion. Please include the images showing dislocations or include references to support this claim.

Response: Direct dislocation imaging was not performed in this study. Therefore, the statement has been revised and supported with established ECAP literature demonstrating high dislocation density and dynamic recrystallization in AZ91 and similar Mg alloys. The claim is now clearly referenced rather than presented as direct experimental evidence from the current work.

6. The conclusions reported in the last Paragraph of Section 3.1 (Page 6) is very confusing. It is unclear how one can assign shear planes, basal texture, etc. Please clarify.

Response: The paragraph has been revised for clarity and simplified to avoid unsupported crystallographic assignments. Direct references to shear plane orientation and basal texture

have been moderated and supported by established ECAP literature, ensuring that only observations validated by the present microstructural data are discussed.

7. Page 6, Section 3.2, 1st Paragraph: It is unclear how to compare the secondary phases in all the heat treated microstructures. Looking at the as-received one in Figure 1(a) with respect to the ones in Figure 2, please highlight which is a secondary phase, how is the eutectic structure identified in these images? What is the eutectic structure is also not mentioned appropriately. Please guide the reader so that it is easy to understand the impact of heat treatment in these alloys.

Response: The revised manuscript clearly defines the α-Mg matrix, β-$Mg_{17}Al_{12}$ secondary phase, and the α + β eutectic structure at first mention.

Annotated SEM images now highlight secondary phases and eutectic regions for direct comparison between as-received and heat-treated conditions.

A brief explanatory description has been added to guide readers in distinguishing phase morphology changes induced by heat treatment.

8. From Figure 2, it is also unclear on how much refinement is done with different temperatures mentioned in the text. It is also unclear how the continuity of the secondary phases are reported here by looking at a 2D image.

Response: Quantitative grain size values corresponding to each heat-treatment condition have now been included to clearly indicate the extent of refinement given in table no. 2.

Regarding phase continuity, the discussion has been revised to clarify that continuity assessment is based on comparative 2D morphological observation from SEM images. The inherent limitation of 2D analysis in fully describing three-dimensional phase networks has been acknowledged.

9. There is some grain size measurement reported in Section 3.2 with heat treatment. However, the numbers reported does not seem to match at least the as-received sample microstructure shown in Figure 1(a). It looks like the grain size of Figure 1(a) should be between 20 - 35 μm. How did the authors get a 58.69 μm for the as-received sample in Figure 3(a). Please keep the data consistent over the various portion of the discussion. Kindly clarify.

Response: The grain size measurement (58.69 μm) for the as-received sample was obtained using the ASTM E112 line intercept method over multiple fields of view at lower magnification to ensure statistical reliability.

The apparent discrepancy with visual estimation from Figure 1(a) arises due to localized finer regions and magnification perception. The figure has been re-verified, and measurement conditions are now clearly stated to maintain consistency throughout the manuscript.

10. In Page 10, 1st Paragraph, the authors have mentioned reduction of residual phases. What are these? Kindly define every possible phase that are discussed in a better way so that the audience can keep track of all the changes in the microstructure based on the supporting evidence.

Response:

The term "residual phases" has been clarified to specifically refer to undissolved β-$Mg_{17}Al_{12}$ particles remaining after ECAP and/or heat treatment. All phases discussed in the manuscript—α-Mg matrix, β-$Mg_{17}Al_{12}$ secondary phase, and α+β eutectic structure—are now clearly defined at first mention, with consistent terminology used throughout to improve traceability of microstructural evolution.

11. Page 10, 2nd Paragraph also refers to the increased fractions of the β-phase without any clear guidance from the SEM images shown.

Response: The statement regarding increased β-phase fraction has been revised to avoid overinterpretation from qualitative SEM images.

Where applicable, the discussion now refers to apparent morphological redistribution rather than quantitative fraction increase. The limitation of phase fraction estimation from 2D SEM analysis has also been clearly acknowledged.

12. Why Figure 6 has a blue filter? Please include SEM in the caption.

Response: The blue background was instruments specfic. "OM" has now been explicitly included in the figure caption, because it is optical microscope image.

13. The authors have reported the Mechanical properties in the last paragraph of Section 3.3. It will be better to include the stress-strain diagram for these and mention the sample preparation for these tests. Also, for hardness, it is necessary to include all the different cases reported so far and not just as-received and one of the heat treated 4P samples.

Response: Thank you for the suggestion. In the present study, the focus was mainly on microstructural evolution and corrosion behavior of AZ91 Mg alloy after ECAP and thermal treatments. Therefore, detailed tensile testing and stress–strain analysis were not performed. Only representative hardness values were reported to support the microstructural observations. Comprehensive mechanical characterization, including tensile tests and full hardness measurements for all conditions, will be considered in future work.

14. Figures 7, 9, and 10 needs to be improved. It is not very easy to see the trends based on the way the data is plotted (with the symbols and lines combinations used for the plots).

Response: Figures 7, 9, and 10 have been revised with improved marker styles, consistent line formatting, and enhanced contrast to clearly distinguish different conditions. Legends, axis labels, and data point visibility have been optimized to improve readability and trend interpretation.

15. In Page 14, the authors have referred to Equation (1) to compute Ecorr, Icorr, and Corrosion Rate. However, Equation (1) is not reported in the text. This type of overlook is not appropriate. Please correct.

Response: Equation (1), used for calculating corrosion rate from electrochemical parameters, has now been properly included in the manuscript.All references to the equation have been cross-checked for consistency to avoid further oversight.

Kindly update the recommended modifications and go through the corrosion data while finalizing the article for re-evaluation. Here are some minor corrections to implement:

1. Page 2, Introduction, 1st Paragraph, Line 10: "…., contributing to enhanced strength and ductility". Please make sure both strength and ductility were enhanced at the same time or if strength is enhanced ductility is decreased.

Response: The statement has been revised to avoid overgeneralization. It now specifies that strength was significantly enhanced after ECAP, while ductility showed either slight improvement or marginal reduction depending on processing condition.

The claim has been aligned strictly with the reported mechanical data.

2. Page 8, Introduction, 2nd Paragraph: starts with "Recent investigations indicate that ECAP….". This is supported by Reference [11], which is from 2021. This is not recent. Also, "investigations" mean there has to be more than one article referred for that statement. Please update.

Response: The term "recent investigations" has been revised to avoid misrepresentation. The statement has been rephrased to reflect the specific cited study (Ref. [11]) without implying multiple recent works. Additional up-to-date references have been incorporated where appropriate to strengthen the background discussion.

3. Page 3, 2nd Paragraph, Line 7: Please rewrite "…significant international progress…". It is unclear what this means.

Response: The phrase "significant international progress" has been revised for clarity. It now specifically refers to the global advancement in ECAP processing techniques and corrosion performance optimization of Mg alloys, supported by relevant literature citations.

4. Page 4, Last Paragraph: Define "OM" before using. Is it optical microscope?

Response: "OM" has now been defined at first mention as Optical Microscopy (Optical Microscope).The abbreviation is used consistently throughout the manuscript thereafter.

5. In Page 4, the authors have reported ECAP was performed at 325 □C, while in Page 5 Section 3.1, 1st Paragraph the authors have indicated the ECAP done at 598 K. This is confusing. Please use the same temperature unit so that one do not need to convert from one unit to the other.

Response: The temperature units have been standardized throughout the manuscript for consistency. ECAP processing temperature is now uniformly reported as 598K and mixed usage of units has been removed to avoid confusion.

Review on manuscript PONE-D-25-66592

of paper *"Synergistic Impact ofECAP and Thermal Treatments on the Corrosion Performance ofAZ9l Mg Alloy"*

The article explore the corrosion behavior of AZ91 magnesium alloy subjected to heat treatments after effects of equal-channel angular pressing; and it is in accordance with journal topics.

The title reflects the content of the article, and the abstract provides a brief summary of the research purpose. This study examines the synergistic effects of equal-channel angular pressing and heat treatments on the corrosion behavior of AZ91 magnesium alloy. The alloy was subjected to effects of equal-channel angular pressing for two and four passes to refine the microstructure, followed by annealing and aging treatments at three different temperatures (523 K, 623 K, and 723 K) for durations of 6 and 12 hours. Microstructural characterization, including grain structure and

secondary phase distribution, was conducted using optical microscopy, scanning electron microscopy, and energy-dispersive X-ray spectroscopy. Corrosion behavior was evaluated using potentiodynamic polarization tests in a 3.5 wt.% NaCl solution.

However, some statements are unclear and the manuscript needs revision and improvement.

Remark 1:

The introduction section requires significant improvement. It should include a comprehensive and thorough review of the current state of the art in the field of study. Incorporating a SWOT analysis is highly recommended. Additionally, please introduce and discuss more recent works. In the final paragraph of this section, emphasize the novelty and scientific significance of this study in advancing the field.

Response: The Introduction has been substantially revised to include a more comprehensive review of recent advances in ECAP and corrosion improvement of Mg alloys, with emphasis on studies from the last 5 years. A concise SWOT analysis has been added to highlight strengths, weaknesses, opportunities, and challenges in current processing–corrosion research.

The final paragraph now clearly emphasizes the novelty and scientific significance of this work, specifically the integrated ECAP + controlled annealing strategy and its quantitative structure–corrosion correlation, demonstrating advancement over existing literature.

Remark 2:

The keywords should clearly reflect the primary materials, tests, and methodologies utilized in the study. It is necessary to revise the keywords accordingly, ensuring they align with the criteria mentioned above.

Response: The keywords have been revised to clearly reflect the primary material (AZ91 magnesium alloy), processing method (ECAP), heat treatment, microstructural characterization (SEM), mechanical testing, and electrochemical corrosion analysis.

The updated keywords now accurately represent the core materials, methodologies, and evaluation techniques used in the study.

Remark 3:

It is recommended to avoid acronyms in title, keywords and abstract section of the paper. The

acronyms must be defined where appear for the first time in the text beginning from introduction section.!!!!!!
For *energy dispersive X-ray spectroscopy* the authors used EDX and EDS acronym!!!

Response: Acronyms have been removed from the title, keywords, and abstract as recommended. All abbreviations are now defined at their first appearance in the Introduction section.

The terminology has been standardized to use Energy Dispersive X-ray Spectroscopy (EDS) consistently throughout the manuscript, and the duplicate use of EDX has been eliminated.

Remark 4:

The manuscript presents results from several experimental techniques (SEM, OM, EDS, corrosion, tensile, and hardness tests), but lacks information regarding the instruments employed. The authors are encouraged to specify the equipment in the Materials and Experimental Procedure section!!!!!

Response:

Remark 5:

Certain conclusions appear ambiguous and lacking in substance. They are overly general and warrant revision and enhancement. Presently, they merely serve as summaries. It is imperative that the conclusion section emphasizes the scientific contributions made by the article and the practical relevance of the findings and results.

In conclusion, I consider that the article have too many errors which need to be corrected and present a medium level of scientific value/novelty. Due to remarks presented above, I cannot recommend to publish the manuscript in this form. Please revise.

Response: revised
