# Supplementary material for: Synergistic impact of ECAP and thermal treatments on the corrosion performance of AZ91 Mg alloy
Source: PLoS One. 2026 Sep 25;21(9):e0354359. doi: 10.1371/journal.pone.0354359 (PMC13614571; doi:10.1371/journal.pone.0354359)
Supplement: Supplementary file 8 [file pone.0354359.s001.docx]

The manuscript presents a relevant topic and contains potentially useful findings. However, in its current form, it requires **major revision** before it can be considered for publication. The primary concerns include an insufficient demonstration of novelty, limited depth in corrosion analysis, a lack of supporting advanced electrochemical characterization, and a largely qualitative rather than quantitative microstructural interpretation. Several conclusions are presented without adequate statistical or mechanistic support. Additional validation methods, more apparent justification of processing parameters, improved image quality, and a deeper mechanistic explanation are required to substantiate the claims. With substantial strengthening of methodology, data interpretation, and presentation clarity, the manuscript may reach publishable quality, but significant development is still needed at this stage.

- The study is interesting, but the novelty could be articulated more clearly to distinguish it from existing works on AZ91 corrosion improvement.
- The manuscript would benefit from a more substantial justification of why the specific ECAP passes and heat-treatment conditions were selected.
- The corrosion analysis is useful but could be strengthened by incorporating additional techniques, such as EIS or immersion testing, to support the polarization results.
- Providing statistical validation or multiple repeat trials would enhance confidence in the reported data.
- The discussion on the synergistic effect is promising but would benefit from clearer evidence or a quantitative demonstration.
- Some claims appear stronger than the supporting data; tempering the language would improve scientific balance.
- The microstructural analysis could be improved with more quantitative metrics, such as grain size distribution or phase fraction estimation.
- Including EBSD or additional crystallographic information may help better explain the recrystallization and texture evolution.
- SEM images are informative, but slightly higher resolution images or clearer contrast may improve visual interpretation.
- The corrosion mechanism explanation could be expanded to provide deeper electrochemical insight and stronger scientific linkage.
- The relationship between β-phase redistribution and corrosion performance would benefit from a more detailed mechanistic explanation.
- More detailed comparison with related recent studies would help highlight where this work stands in the broader research landscape.
- A clearer separation between observation and interpretation in the results section would improve readability.
- Adding a brief limitations section could help provide perspective and transparency to the study.
- Figures are useful, but more consistent annotation formatting would enhance presentation quality.
- The narrative flow could be slightly tightened in places to make the discussion more concise and impactful.
- Including a discussion of long-term stability or environmental relevance would increase the practical value of the findings.
- Some sections could benefit from clearer transitions to guide the reader logically through the results.
- The conclusion effectively summarizes the findings but could be more explicitly linked to industrial or application relevance.
- Overall, the work shows promise and contains meaningful observations. With some strengthening of analysis and presentation, it has good potential.
