# Supplementary material for: Synergistic impact of ECAP and thermal treatments on the corrosion performance of AZ91 Mg alloy
Source: PLoS One. 2026 Sep 25;21(9):e0354359. doi: 10.1371/journal.pone.0354359 (PMC13614571; doi:10.1371/journal.pone.0354359)
Supplement: Supplementary file 9 [file pone.0354359.s005.docx]

I have reviewed the revised version of the manuscript titled "Synergistic Impact of ECAP and Thermal Treatments on the Corrosion Performance of AZ91 Mg Alloy." The authors have carefully addressed all of the reviewers' comments, including revisions to the experimental data, improvements in figure clarity, and a more thorough discussion of the experimental results. The revised manuscript is now better, with clearer explanations of the novelty, the synergistic effects of ECAP and heat treatment, and the statistical validation of the data.

After reviewing the manuscript and the authors' responses, I am pleased to inform you that we are satisfied with the revisions. Therefore, I would like to formally accept the manuscript for publication.
