# Supplementary material for: Synergistic impact of ECAP and thermal treatments on the corrosion performance of AZ91 Mg alloy
Source: PLoS One. 2026 Sep 25;21(9):e0354359. doi: 10.1371/journal.pone.0354359 (PMC13614571; doi:10.1371/journal.pone.0354359)
Supplement: Peer review history

# Peer review: Decision Letter 0

**DOI:** 10.1371/journal.pone.0354359.r001

**Authors:** Kuldeep Kumar Saxena

20 Jan 2026

Dear Dr. Anne,

Thank you for submitting your manuscript to PLOS ONE. After careful consideration, we feel that it has merit but does not fully meet PLOS ONE’s publication criteria as it currently stands. Therefore, we invite you to submit a revised version of the manuscript that addresses the points raised during the review process.

Please submit your revised manuscript by Mar 06 2026 11:59PM. If you will need more time than this to complete your revisions, please reply to this message or contact the journal office at plosone@plos.org. When you're ready to submit your revision, log on to https://www.editorialmanager.com/pone/ and select the 'Submissions Needing Revision' folder to locate your manuscript file.

- A letter that responds to each point raised by the academic editor and reviewer(s). You should upload this letter as a separate file labeled 'Response to Reviewers'.
- A marked-up copy of your manuscript that highlights changes made to the original version. You should upload this as a separate file labeled 'Revised Manuscript with Track Changes'.
- An unmarked version of your revised paper without tracked changes. You should upload this as a separate file labeled 'Manuscript'.

If you would like to make changes to your financial disclosure, please include your updated statement in your cover letter. Guidelines for resubmitting your figure files are available below the reviewer comments at the end of this letter.

If applicable, we recommend that you deposit your laboratory protocols in protocols.io to enhance the reproducibility of your results. Protocols.io assigns your protocol its own identifier (DOI) so that it can be cited independently in the future. For instructions see: https://journals.plos.org/plosone/s/submission-guidelines#loc-laboratory-protocols. Additionally, PLOS ONE offers an option for publishing peer-reviewed Lab Protocol articles, which describe protocols hosted on protocols.io. Read more information on sharing protocols at https://plos.org/protocols?utm_medium=editorial-email&utm_source=authorletters&utm_campaign=protocols.

We look forward to receiving your revised manuscript.

Kind regards,

Kuldeep Kumar Saxena

Academic Editor

PLOS One

Journal Requirements:

When submitting your revision, we need you to address these additional requirements.

1. Please ensure that your manuscript meets PLOS ONE's style requirements, including those for file naming. The PLOS ONE style templates can be found at

https://journals.plos.org/plosone/s/file?id=wjVg/PLOSOne_formatting_sample_main_body.pdf and

https://journals.plos.org/plosone/s/file?id=ba62/PLOSOne_formatting_sample_title_authors_affiliations.pdf

2. Please update your submission to use the PLOS LaTeX template. The template and more information on our requirements for LaTeX submissions can be found at http://journals.plos.org/plosone/s/latex.

3. Thank you for stating the following in the Acknowledgments Section of your manuscript:

“The authors did not receive support from any organization for the submitted work.”

We note that you have provided funding information that is not currently declared in your Funding Statement. However, funding information should not appear in the Acknowledgments section or other areas of your manuscript. We will only publish funding information present in the Funding Statement section of the online submission form.

Please remove any funding-related text from the manuscript and let us know how you would like to update your Funding Statement. Currently, your Funding Statement reads as follows:

“The author(s) received no specific funding for this work.”

Please include your amended statements within your cover letter; we will change the online submission form on your behalf.

4. We note that your Data Availability Statement is currently as follows: “If the data are all contained within the manuscript and/or Supporting Information files, enter the following: All relevant data are within the manuscript and its Supporting Information files.”

Please confirm at this time whether or not your submission contains all raw data required to replicate the results of your study. Authors must share the “minimal data set” for their submission. PLOS defines the minimal data set to consist of the data required to replicate all study findings reported in the article, as well as related metadata and methods (https://journals.plos.org/plosone/s/data-availability#loc-minimal-data-set-definition).

For example, authors should submit the following data:

- The values behind the means, standard deviations and other measures reported;

- The values used to build graphs;

- The points extracted from images for analysis.

Authors do not need to submit their entire data set if only a portion of the data was used in the reported study.

If your submission does not contain these data, please either upload them as Supporting Information files or deposit them to a stable, public repository and provide us with the relevant URLs, DOIs, or accession numbers. For a list of recommended repositories, please see https://journals.plos.org/plosone/s/recommended-repositories.

If there are ethical or legal restrictions on sharing a de-identified data set, please explain them in detail (e.g., data contain potentially sensitive information, data are owned by a third-party organization, etc.) and who has imposed them (e.g., an ethics committee). Please also provide contact information for a data access committee, ethics committee, or other institutional body to which data requests may be sent. If data are owned by a third party, please indicate how others may request data access.

5. PLOS requires an ORCID iD for the corresponding author in Editorial Manager on papers submitted after December 6th, 2016. Please ensure that you have an ORCID iD and that it is validated in Editorial Manager. To do this, go to ‘Update my Information’ (in the upper left-hand corner of the main menu), and click on the Fetch/Validate link next to the ORCID field. This will take you to the ORCID site and allow you to create a new iD or authenticate a pre-existing iD in Editorial Manager.

6. Please amend the manuscript submission data (via Edit Submission) to include author “Ravi Kumar S. R.”

7. If the reviewer comments include a recommendation to cite specific previously published works, please review and evaluate these publications to determine whether they are relevant and should be cited. There is no requirement to cite these works unless the editor has indicated otherwise.

[Note: HTML markup is below. Please do not edit.]

Reviewers' comments:

Reviewer's Responses to Questions

**Comments to the Author**

1. Is the manuscript technically sound, and do the data support the conclusions?

Reviewer #1: Partly

Reviewer #2: Yes

Reviewer #3: No

**********

2. Has the statistical analysis been performed appropriately and rigorously?

Reviewer #1: Yes

Reviewer #2: Yes

Reviewer #3: Yes

**********

3. Have the authors made all data underlying the findings in their manuscript fully available?

The PLOS Data policy

Reviewer #1: Yes

Reviewer #2: Yes

Reviewer #3: No

**********

4. Is the manuscript presented in an intelligible fashion and written in standard English?

Reviewer #1: Yes

Reviewer #2: Yes

Reviewer #3: No

**********

Reviewer #1: The manuscript presents a relevant topic and contains potentially useful findings. However, in its current form, it requires major revision before it can be considered for publication. The primary concerns include an insufficient demonstration of novelty, limited depth in corrosion analysis, a lack of supporting advanced electrochemical characterization, and a largely qualitative rather than quantitative microstructural interpretation. Several conclusions are presented without adequate statistical or mechanistic support. Additional validation methods, more apparent justification of processing parameters, improved image quality, and a deeper mechanistic explanation are required to substantiate the claims. With substantial strengthening of methodology, data interpretation, and presentation clarity, the manuscript may reach publishable quality, but significant development is still needed at this stage.

• The study is interesting, but the novelty could be articulated more clearly to distinguish it from existing works on AZ91 corrosion improvement.

• The manuscript would benefit from a more substantial justification of why the specific ECAP passes and heat-treatment conditions were selected.

• The corrosion analysis is useful but could be strengthened by incorporating additional techniques, such as EIS or immersion testing, to support the polarization results.

• Providing statistical validation or multiple repeat trials would enhance confidence in the reported data.

• The discussion on the synergistic effect is promising but would benefit from clearer evidence or a quantitative demonstration.

• Some claims appear stronger than the supporting data; tempering the language would improve scientific balance.

• The microstructural analysis could be improved with more quantitative metrics, such as grain size distribution or phase fraction estimation.

• Including EBSD or additional crystallographic information may help better explain the recrystallization and texture evolution.

• SEM images are informative, but slightly higher resolution images or clearer contrast may improve visual interpretation.

• The corrosion mechanism explanation could be expanded to provide deeper electrochemical insight and stronger scientific linkage.

• The relationship between β-phase redistribution and corrosion performance would benefit from a more detailed mechanistic explanation.

• More detailed comparison with related recent studies would help highlight where this work stands in the broader research landscape.

• A clearer separation between observation and interpretation in the results section would improve readability.

• Adding a brief limitations section could help provide perspective and transparency to the study.

• Figures are useful, but more consistent annotation formatting would enhance presentation quality.

• The narrative flow could be slightly tightened in places to make the discussion more concise and impactful.

• Including a discussion of long-term stability or environmental relevance would increase the practical value of the findings.

• Some sections could benefit from clearer transitions to guide the reader logically through the results.

• The conclusion effectively summarizes the findings but could be more explicitly linked to industrial or application relevance.

• Overall, the work shows promise and contains meaningful observations. With some strengthening of analysis and presentation, it has good potential.

Reviewer #2: The article explore the corrosion behavior of AZ91 magnesium alloy subjected to heat treatments after effects of equal-channel angular pressing; and it is in accordance with journal topics.

Dear authors, please see the remarks presented in the attached review document.

Reviewer #3: Please check the attached document

**********

what does this mean?). If published, this will include your full peer review and any attached files.

If you choose “no”, your identity will remain anonymous but your review may still be made public.

**Do you want your identity to be public for this peer review?**  For information about this choice, including consent withdrawal, please see our Privacy Policy

Reviewer #1: No

Reviewer #2: No

Reviewer #3: No

**********

[NOTE: If reviewer comments were submitted as an attachment file, they will be attached to this email and accessible via the submission site. Please log into your account, locate the manuscript record, and check for the action link "View Attachments". If this link does not appear, there are no attachment files.]

To ensure your figures meet our technical requirements, please review our figure guidelines: https://journals.plos.org/plosone/s/figures

You may also use PLOS’s free figure tool, NAAS, to help you prepare publication quality figures: https://journals.plos.org/plosone/s/figures#loc-tools-for-figure-preparation.

NAAS will assess whether your figures meet our technical requirements by comparing each figure against our figure specifications.

Attachment Submitted filename: review comments.docx

Attachment Submitted filename: Review-PONE-D-25-66592.pdf

Attachment Submitted filename: PONE-D-25-66592_review.pdf

# Author comment: Author response to Decision Letter 1

**DOI:** 10.1371/journal.pone.0354359.r002

9 Mar 2026

The manuscript presents a relevant topic and contains potentially useful findings. However, in its current form, it requires major revision before it can be considered for publication. The primary concerns include an insufficient demonstration of novelty, limited depth in corrosion analysis, a lack of supporting advanced electrochemical characterization, and a largely qualitative rather than quantitative microstructural interpretation. Several conclusions are presented without adequate statistical or mechanistic support. Additional validation methods, more apparent justification of processing parameters, improved image quality, and a deeper mechanistic explanation are required to substantiate the claims. With substantial strengthening of methodology, data interpretation, and presentation clarity, the manuscript may reach publishable quality, but significant development is still needed at this stage.

• The study is interesting, but the novelty could be articulated more clearly to distinguish it from existing works on AZ91 corrosion improvement.

Response: The novelty lies in the systematic integration of ECAP (2P and 4P) with controlled annealing treatments (523–723 K) to establish a direct quantitative correlation between grain refinement, β-Mg_1_₇Al_1__2_ redistribution, and corrosion performance in AZ91 alloy. Unlike prior studies that examined ECAP or heat treatment independently, this work demonstrates the synergistic effect of combined processing, achieving ~99% reduction in corrosion rate and providing structure–corrosion relationships under identical processing conditions. Mentioned in introduction section

• The manuscript would benefit from a more substantial justification of why the specific ECAP passes and heat-treatment conditions were selected.

Response: The Introduction has been revised to explicitly differentiate this work from prior studies by highlighting the combined ECAP + controlled annealing approach and its quantitative corrosion improvement.

The heat-treatment temperatures (523–723 K) were chosen based on the precipitation and stability range of the β-Mg_1_₇Al_1__2_ phase. Specifically, 523 K promotes initial precipitation, 623 K enables redistribution and coarsening, and 723 K approaches homogenization without reaching the incipient melting temperature.

• The corrosion analysis is useful but could be strengthened by incorporating additional techniques, such as EIS or immersion testing, to support the polarization results.

Response: Potentiodynamic polarization was selected to quantitatively determine Ecorr, Icorr, and corrosion rate, enabling direct comparison between processing conditions. While techniques such as EIS or long-term immersion testing could provide additional insight into film stability and degradation kinetics, the polarization results are internally consistent and supported by SEM corrosion morphology analysis.The absence of EIS/immersion testing is acknowledged as a limitation and suggested for future work.

• Providing statistical validation or multiple repeat trials would enhance confidence in the reported data.

Response: All electrochemical tests were performed in triplicate under identical conditions, and the reported values represent averaged results. The experimental deviation was within acceptable limits for corrosion testing of magnesium alloys. This clarification has been added to the methodology section to strengthen statistical reliability.

• The discussion on the synergistic effect is promising but would benefit from clearer evidence or a quantitative demonstration.

Response: The synergistic effect is quantitatively demonstrated through the combined reduction in grain size (58.69 µm → 7.58 µm) and corrosion current density (0.2630 → 0.0011 mA/cm²), resulting in ~99% reduction in corrosion rate. This simultaneous microstructural refinement and β-Mg_1_₇Al_1__2_ redistribution confirms that corrosion improvement arises from the integrated ECAP–annealing treatment rather than individual processing steps.

• Some claims appear stronger than the supporting data; tempering the language would improve scientific balance.

Response: The manuscript has been revised to moderate strong or generalized statements. Conclusions are now strictly aligned with experimentally measured data (Ecorr, Icorr, grain size, hardness).

• The microstructural analysis could be improved with more quantitative metrics, such as grain size distribution or phase fraction estimation.

Response: Quantitative grain size measurements were performed using the ASTM E112 line intercept method and average grain size values are now reported for all key conditions.

Additionally, relative β-Mg_1_₇Al_1__2_ phase fraction trends were discussed based on comparative SEM analysis. The manuscript has been revised to incorporate these quantitative metrics to strengthen microstructural interpretation.

• Including EBSD or additional crystallographic information may help better explain the recrystallization and texture evolution.

Response: While EBSD would provide detailed crystallographic texture and recrystallization analysis, the present study focused primarily on microstructure–corrosion correlation. Grain refinement and phase redistribution were validated using OM and SEM observations.

The absence of EBSD characterization is acknowledged as a limitation and detailed texture analysis is proposed as part of future work.

• SEM images are informative, but slightly higher resolution images or clearer contrast may improve visual interpretation.

Response: Higher-resolution SEM images with improved contrast have been included in the revised manuscript. Image annotations and scale bars were also enhanced to improve phase visibility and overall microstructural interpretation.

• The corrosion mechanism explanation could be expanded to provide deeper electrochemical insight and stronger scientific linkage.

Response: The corrosion mechanism discussion has been expanded to clarify the galvanic interaction between the α-Mg matrix (anodic) and β-Mg_1_₇Al_1__2_ phase (cathodic). The role of grain refinement in promoting uniform corrosion, reducing localized galvanic coupling, and stabilizing the surface Mg(OH)_2_ film has been explained to provide stronger electrochemical linkage with the polarization data.

• The relationship between β-phase redistribution and corrosion performance would benefit from a more detailed mechanistic explanation.

Response: The manuscript now clarifies that in the as-received condition, β-Mg_1_₇Al_1__2_ forms a continuous network along grain boundaries, acting as strong cathodic sites and promoting localized galvanic corrosion. After ECAP and annealing, the β-phase becomes finer and more uniformly dispersed within the α-Mg matrix, reducing cathodic intensity, interrupting galvanic continuity and promoting more homogeneous corrosion. This redistribution lowers localized pit initiation and decreases overall corrosion current density, directly linking phase morphology to improved corrosion performance.

• More detailed comparison with related recent studies would help highlight where this work stands in the broader research landscape.

Response: A concise comparison has been added summarizing corrosion rates, processing methods (ECAP/annealing/other SPD), and microstructure–property relationships from recent studies on similar Mg alloys. This comparison highlights how the current integrated ECAP + controlled annealing approach achieves superior corrosion reduction and provides clearer structure–performance linkage compared to individual processing strategies reported in the literature.

• A clearer separation between observation and interpretation in the results section would improve readability.

Response: The Results section has been reorganized to clearly distinguish experimental observations (micrographs, measured values, polarization data) from mechanistic interpretation. Descriptive findings are now presented first, followed by a separate analytical discussion to improve clarity and scientific readability.

• Adding a brief limitations section could help provide perspective and transparency to the study.

Response: A dedicated limitations section has been incorporated, noting the absence of EBSD-based texture analysis, EIS/long-term immersion validation, and precise quantitative phase fraction measurements. These factors are recognized as scope boundaries of the present work and are proposed as future extensions to further reinforce the microstructure–corrosion relationship.

• Figures are useful, but more consistent annotation formatting would enhance presentation quality.

Response: All figures have been reformatted to ensure consistent font size, labeling style, arrow markings and scale bar presentation. Captions were standardized and phase annotations were uniformly applied to improve clarity and overall presentation quality.

• The narrative flow could be slightly tightened in places to make the discussion more concise and impactful.

Response: The manuscript has been carefully edited to remove redundant statements and streamline transitions between sections. The discussion has been tightened to focus on key quantitative findings and mechanistic insights, improving clarity and overall impact.

• Including a discussion of long-term stability or environmental relevance would increase the practical value of the findings.

Response: A brief discussion on long-term corrosion stability and environmental relevance has been added. The potential applicability of the optimized ECAP + annealing condition for chloride-rich environments such as marine, aerosace and automotive exposure is highlighted, while noting that extended immersion studies are recommended for future validation.

• Some sections could benefit from clearer transitions to guide the reader logically through the results.

Response: Transitional sentences have been added between microstructural characterization, mechanical properties and corrosion analysis sections to improve logical continuity. The revised structure now guides the reader sequentially from processing, microstructure evolution and corrosion performance.

• The conclusion effectively summarizes the findings but could be more explicitly linked to industrial or application relevance.

Response: The conclusion has been revised to explicitly link the improved corrosion resistance and mechanical strengthening to lightweight structural applications in automotive, aerospace, and marine environments. The practical significance of the optimized ECAP + annealing condition is now clearly stated in terms of enhanced durability and service life in chloride-containing conditions.

• Overall, the work shows promise and contains meaningful observations. With some strengthening of analysis and presentation, it has good potential.

Response: We appreciate the positive assessment. The manuscript has been substantially strengthened through improved quantitative analysis, clearer mechanistic explanation, enhanced figure quality and moderated conclusions.

Review on “Synergistic Impact of ECAP and Thermal Treatments on the Corrosion Performance of AZ91 Mg Alloy”

The authors have attempted to systematically investigate the mechanical and corrosion behavior of AZ91 Mg alloy by performing ECAP 2P, 4P and heat treatment at various temperatures and time. While the work is interesting for PLOS One, there is a lot of scientific issues that are unclear and needs clarification before consideration for publication. The referee recommends the work to be considered for publication after major revision. Here are some of the points the authors must address for future consideration:

1. Please guide the readers by clearly marking the secondary phases, eutectic phases, residual phases or any other phases in a microstructure before describing the details.

Response: All microstructures have been revised with clear annotations identifying the α-Mg matrix, β-Mg_1_₇Al_1__2_ secondary phase, eutectic (α + β) regions, and residual particles. Arrows and labeled insets are added in the figures and phase descriptions are clarified in the captions prior to detailed discussion in the text.

2. In many instances, the authors have mentioned that the corrosion resistance is improved in Section 3 without any supporting data. Please conclude only if you have supporting evidence or wait till the evidence is shown to conclude on corrosion resistance.

Response: The manuscript has been revised to ensure that all statements regarding corrosion resistance are made only after presenting supporting electrochemical data (Ecorr, Icorr, and corrosion rate).Premature conclusions have been removed and interpretations are now strictly aligned with the corresponding experimental evidence.

3. Figure 1 shows the SEM image of the as-received, 2P and 4P microstructures. While the as-received microstructure is shown at x1000, the other two are shown at a higher magnification. It is generally better to show the same magnification is available. While the scale bar should be used to analyze the images independently, since these are shown side-by-side, a quick glance may indicate that the grain sizes are the similar for all cases. If the authors want to keep the same images, please include an average ASTM grain size calculated for all the three cases both in the Figure caption and in the text for comparison. This will support the conclusion in the last Paragraph of Page 5 on grain refinement due to 2P and 4P with quantitative numbers.

Response: Figure 1 has been revised to ensure consistent magnification for direct comparison. Additionally, average grain size values measured using the ASTM E112 line intercept method are now included in both the figure caption and text (as-received: 58.69 µm; 2P: 30.86 µm; 4P: 7.58 µm). This quantitative comparison clearly supports the conclusion on progressive grain refinement after 2P and 4P ECAP processing

4. In Figure 1(a), the claim of coarse-grained structure is accepted, but it is better to quantify it with the comment in 1. In addition, the claim that there are high fraction of secondary phases along the grain boundaries are not clear at all. Please highlight the areas or provide supporting evidence of this grain boundary segregation.

Response: The coarse-grained structure in Figure 1(a) is now quantitatively supported with the measured average grain size (58.69 µm) using the ASTM E112 method.Grain boundary β-Mg_1_₇Al_1__2_ segregation has been highlighted with annotated regions in the SEM image, and compositional confirmation from EDS analysis is referenced to support phase identification.

5. In Page 5, last paragraph, the authors have reported that ECAP generates high density of dislocations and activates dynamic recrystallization. There is no supporting data shown for this conclusion. Please include the images showing dislocations or include references to support this claim.

Response: Direct dislocation imaging was not performed in this study. Therefore, the statement has been revised and supported with established ECAP literature demonstrating high dislocation density and dynamic recrystallization in AZ91 and similar Mg alloys. The claim is now clearly referenced rather than presented as direct experimental evidence from the current work.

6. The conclusions reported in the last Paragraph of Section 3.1 (Page 6) is very confusing. It is unclear how one can assign shear planes, basal texture, etc. Please clarify.

Response: The paragraph has been revised for clarity and simplified to avoid unsupported crystallographic assignments. Direct references to shear plane orientation and basal texture have been moderated and supported by established ECAP literature, ensuring that only observations validated by the present microstructural data are discussed.

7. Page 6, Section 3.2, 1st Paragraph: It is unclear how to compare the secondary phases in all the heat treated microstructures. Looking at the as-received one in Figure 1(a) with respect to the ones in Figure 2, please highlight which is a secondary phase, how is the eutectic structure identified in these images? What is the eutectic structure is also not mentioned appropriately. Please guide the reader so that it is easy to understand the impact of heat treatment in these alloys.

Response:The revised manuscript clearly defines the α-Mg matrix, β-Mg_1_₇Al_1__2_ secondary phase, and the α + β eutectic structure at first mention.

Annotated SEM images now highlight secondary phases and eutectic regions for direct comparison between as-received and heat-treated conditions.

A brief explanatory description has been added to guide readers in distinguishing phase morphology changes induced by heat treatment.

8. From Figure 2, it is also unclear on how much refinement is done with different temperatures mentioned in the text. It is also unclear how the continuity of the secondary phases are reported here by looking at a 2D image.

Response: Quantitative grain size values corresponding to each heat-treatment condition have now been included to clearly indicate the extent of refinement given in table no. 2.

Regarding phase continuity, the discussion has been revised to clarify that continuity assessment is based on comparative 2D morphological observation from SEM images. The inherent limitation of 2D analysis in fully describing three-dimensional phase networks has been acknowledged.

9. There is some grain size measurement reported in Section 3.2 with heat treatment. However, the numbers reported does not seem to match at least the as-received sample microstructure shown in Figure 1(a). It looks like the grain size of Figure 1(a) should be between 20 - 35 µm. How did the authors get a 58.69 µm for the as-received sample in Figure 3(a). Please keep the data consistent over the various portion of the discussion. Kindly clarify.

Response: The grain size measurement (58.69 µm) for the as-received sample was obtained using the ASTM E112 line intercept method over multiple fields of view at lower magnification to ensure statistical reliability.

The apparent discrepancy with visual estimation from Figure 1(a) arises due to localized finer regions and magnification perception. The figure has been re-verified, and measurement conditions are now clearly stated to maintain consistency throughout the manuscript.

10. In Page 10, 1st Paragraph, the authors have mentioned reduction of residual phases. What are these? Kindly define every possible phase that are discussed in a better way so that the audience can keep track of all the changes in the microstructure based on the supporting evidence.

Response:

The term “residual phases” has been clarified to specifically refer to undissolved β-Mg_1_₇Al_1__2_ particles remaining after ECAP and/or heat treatment. All phases discussed in the manuscript—α-Mg matrix, β-Mg_1_₇Al_1__2_ secondary phase, and α+β eutectic structure—are now clearly defined at first mention, with consistent terminology used throughout to improve traceability of microstructural evolution.

11. Page 10, 2nd Paragraph also refers to the increased fractions of the β-phase without any clear guidance from the SEM images shown.

Response: The statement regarding increased β-phase fraction has been revised to avoid overinterpretation from qualitative SEM images.

Where applicable, the discussion now refers to apparent morphological redistribution rather than quantitative fraction increase. The limitation of phase fraction estimation from 2D SEM analysis has also been clearly acknowledged.

12. Why Figure 6 has a blue filter? Please include SEM in the caption.

Response: The blue background was instruments specfic. “OM” has now been explicitly included in the figure caption, because it is optical microscope image.

13. The authors have reported the Mechanical properties in the last paragraph of Section 3.3. It will be better to include the stress-strain diagram for these and mention the sample preparation for these tests. Also, for hardness, it is necessary to include all the different cases reported so far and not just as-received and one of the heat treated 4P samples.

Response: Thank you for the suggestion. In the present study, the focus was mainly on microstructural evolution and corrosion behavior of AZ91 Mg alloy after ECAP and thermal treatments. Therefore, detailed tensile testing and stress–strain analysis were not performed. Only representative hardness values were reported to support the microstructural observations. Comprehensive mechanical characterization, including tensile tests and full hardness measurements for all conditions, will be considered in future work.

14. Figures 7, 9, and 10 needs to be improved. It is not very easy to see the trends based on the way the data is plotted (with the symbols and lines combinations used for the plots).

Response: Figures 7, 9, and 10 have been revised with improved marker styles, consistent line formatting, and enhanced contrast to clearly distinguish different conditions. Legends, axis labels, and data point visibility have been optimized to improve readability and trend interpretation.

15. In Page 14, the authors have referred to Equation (1) to compute Ecorr, Icorr, and Corrosion Rate. However, Equation (1) is not reported in the text. This type of overlook is not appropriate. Please correct.

Response: Equation (1), used for calculating corrosion rate from electrochemical parameters, has now been properly included in the manuscript.All references to the equation have been cross-checked for consistency to avoid further oversight.

Kindly update the recommended modifications and go through the corrosion data while finalizing the article for re-evaluation. Here are some minor corrections to implement:

1. Page 2, Introduction, 1st Paragraph, Line 10: “…., contributing to enhanced strength and ductility”. Please make sure both strength and ductility were enhanced at the same time or if strength is enhanced ductility is decreased.

Response: The statement has been revised to avoid overgeneralization. It now specifies that strength was significantly enhanced after ECAP, while ductility showed either slight improvement or marginal reduction depending on processing condition.

The claim has been aligned strictly with the reported mechanical data.

2. Page 8, Introduction, 2nd Paragraph: starts with “Recent investigations indicate that ECAP….”. This is supported by Reference [11], which is from 2021. This is not recent. Also, “investigations” mean there has to be more than one article referred for that statement. Please update.

Response: The term “recent investigations” has been revised to avoid misrepresentation. The statement has been rephrased to reflect the specific cited study (Ref. [11]) without implying multiple recent works. Additional up-to-date references have been incorporated where appropriate to strengthen the background discussion.

3. Page 3, 2nd Paragraph, Line 7: Please rewrite “…significant international progress…”. It is unclear what this means.

Response: The phrase “significant international progress” has been revised for clarity. It now specifically refers to the global advancement in ECAP processing techniques and corrosion performance optimization of Mg alloys, supported by relevant literature citations.

4. Page 4, Last Paragraph: Define “OM” before using. Is it optical microscope?

Response: “OM” has now been defined at first mention as Optical Microscopy (Optical Microscope).The abbreviation is used consistently throughout the manuscript thereafter.

5. In Page 4, the authors have reported ECAP was performed at 325 °C, while in Page 5 Section 3.1, 1st Paragraph the authors have indicated the ECAP done at 598 K. This is confusing. Please use the same temperature unit so that one do not need to convert from one unit to the other.

Response: The temperature units have been standardized throughout the manuscript for consistency. ECAP processing temperature is now uniformly reported as 598K and mixed usage of units has been removed to avoid confusion.

Review on manuscript PONE-D-25-66592

of paper “Synergistic Impact ofECAP and Thermal Treatments on the Corrosion Performance ofAZ9l Mg Alloy”

The article explore the corrosion behavior of AZ91 magnesium alloy subjected to heat treatments after effects of equal-channel angular pressing; and it is in accordance with journal topics.

The title reflects the content of the article, and the abstract provides a brief summary of the research purpose. This study examines the synergistic effects of equal-channel angular pressing and heat treatments on the corrosion behavior of AZ91 magnesium alloy. The alloy was subjected to effects of equal-channel angular pressing for two and four passes to refine the microstructure, followed by annealing and aging treatments at three different temperatures (523 K, 623 K, and 723 K) for durations of 6 and 12 hours. Microstructural characterization, including grain structure and secondary phase distribution, was conducted using optical microscopy, scanning electron microscopy, and energy-dispersive X-ray spectroscopy. Corrosion behavior was evaluated using potentiodynamic polarization tests in a 3.5 wt.% NaCl solution.

However, some statements are unclear and the manuscript needs revision and improvement.

Remark 1:

The introduction section requires significant improvement. It should include a comprehensive and thorough review of the current state of the art in the field of study. Incorporating a SWOT analysis is highly recommended. Additionally, please introduce and discuss more recent works. In the final paragraph of this section, emphasize the novelty and scientific significance of this study in advancing the field.

Response: The Introduction has been substantially revised to include a more comprehensive review of recent advances in ECAP and corrosion improvement of Mg alloys, with emphasis on studies from the last 5 years. A concise SWOT analysis has been added to highlight strengths, weaknesses, opportunities, and challenges in current processing–corrosion research.

The final paragraph now clearly emphasizes the novelty and scientific significance of this work, specifically the integrated ECAP + controlled annealing strategy and its quantitative structure–corrosion correlation, demonstrating advancement over existing literature.

Remark 2:

The keywords should clearly reflect the primary materials, tests, and methodologies utilized in the study. It is necessary to revise the keywords accordingly, ensuring they align with the criteria mentioned above.

Response: The keywords have been revised to clearly reflect the primary material (AZ91 magnesium alloy), processing method (ECAP), heat treatment, microstructural characterization (SEM), mechanical testing, and electrochemical corrosion analysis.

The updated keywords now accurately represent the core materials, methodologies, and evaluation techniques used in the study.

Remark 3:

It is recommended to avoid acronyms in title, keywords and abstract section of the paper. The acronyms must be defined where appear for the first time in the text beginning from introduction section.!!!!!!

For energy dispersive X-ray spectroscopy the authors used EDX and EDS acronym! !!

Response: Acronyms have been removed from the title, keywords, and abstract as recommended. All abbreviations are now defined at their first appearance in the Introduction section.

The terminology has been standardized to use Energy Dispersive X-ray Spectroscopy (EDS) consistently throughout the manuscript, and the duplicate use of EDX has been eliminated.

Remark 4:

The manuscript presents results from several experimental techniques (SEM, OM, EDS, corrosion, tensile, and hardness tests), but lacks information regarding the instruments employed. The authors are encouraged to specify the equipment in the Materials and Experimental Procedure section! !!!!!

Response:

Remark 5:

Certain conclusions appear ambiguous and lacking in substance. They are overly general and warrant revision and enhancement. Presently, they merely serve as summaries. It is imperative that the conclusion section emphasizes the scientific contributions made by the article and the practical relevance of the findings and results.

In conclusion, I consider that the article have too many errors which need to be corrected and present a medium level of scientific value/novelty. Due to remarks presented above, I cannot recommend to publish the manuscript in this form. Please revise.

Response: revised

Attachment Submitted filename: Answar to Reviewer Comments.pdf

# Peer review: Decision Letter 1

**DOI:** 10.1371/journal.pone.0354359.r003

**Authors:** Kuldeep Kumar Saxena

20 Apr 2026

Dear Dr. Anne,

Thank you for submitting your manuscript to PLOS ONE. After careful consideration, we feel that it has merit but does not fully meet PLOS ONE’s publication criteria as it currently stands. Therefore, we invite you to submit a revised version of the manuscript that addresses the points raised during the review process.

Please submit your revised manuscript by Jun 04 2026 11:59PM. If you will need more time than this to complete your revisions, please reply to this message or contact the journal office at plosone@plos.org. When you're ready to submit your revision, log on to https://www.editorialmanager.com/pone/ and select the 'Submissions Needing Revision' folder to locate your manuscript file.

- A letter that responds to each point raised by the academic editor and reviewer(s). You should upload this letter as a separate file labeled 'Response to Reviewers'.
- A marked-up copy of your manuscript that highlights changes made to the original version. You should upload this as a separate file labeled 'Revised Manuscript with Track Changes'.
- An unmarked version of your revised paper without tracked changes. You should upload this as a separate file labeled 'Manuscript'.

If you would like to make changes to your financial disclosure, please include your updated statement in your cover letter. Guidelines for resubmitting your figure files are available below the reviewer comments at the end of this letter.

If applicable, we recommend that you deposit your laboratory protocols in protocols.io to enhance the reproducibility of your results. Protocols.io assigns your protocol its own identifier (DOI) so that it can be cited independently in the future. For instructions see: https://journals.plos.org/plosone/s/submission-guidelines#loc-laboratory-protocols. Additionally, PLOS ONE offers an option for publishing peer-reviewed Lab Protocol articles, which describe protocols hosted on protocols.io. Read more information on sharing protocols at https://plos.org/protocols?utm_medium=editorial-email&utm_source=authorletters&utm_campaign=protocols.

As the corresponding author, your ORCID iD is verified in the submission system and will appear in the published article. PLOS supports the use of ORCID, and we encourage all coauthors to register for an ORCID iD and use it as well. Please encourage your coauthors to verify their ORCID iD within the submission system before final acceptance, as unverified ORCID iDs will not appear in the published article. *Only*  the individual author can complete the verification step; PLOS staff *cannot*  verify ORCID iDs on behalf of authors.

We look forward to receiving your revised manuscript.

Kind regards,

Kuldeep Kumar Saxena

Academic Editor

PLOS One

Journal Requirements:

1. If the reviewer comments include a recommendation to cite specific previously published works, please review and evaluate these publications to determine whether they are relevant and should be cited. There is no requirement to cite these works unless the editor has indicated otherwise.

2. Please review your reference list to ensure that it is complete and correct. If you have cited papers that have been retracted, please include the rationale for doing so in the manuscript text, or remove these references and replace them with relevant current references. Any changes to the reference list should be mentioned in the rebuttal letter that accompanies your revised manuscript. If you need to cite a retracted article, indicate the article’s retracted status in the References list and also include a citation and full reference for the retraction notice.

[Note: HTML markup is below. Please do not edit.]

Reviewers' comments:

Reviewer's Responses to Questions

**Comments to the Author**

Reviewer #1: All comments have been addressed

Reviewer #2: All comments have been addressed

Reviewer #3: (No Response)

**********

2. Is the manuscript technically sound, and do the data support the conclusions?

Reviewer #1: Yes

Reviewer #2: Yes

Reviewer #3: Yes

**********

3. Has the statistical analysis been performed appropriately and rigorously?

Reviewer #1: Yes

Reviewer #2: Yes

Reviewer #3: No

**********

4. Have the authors made all data underlying the findings in their manuscript fully available?

The PLOS Data policy

Reviewer #1: Yes

Reviewer #2: Yes

Reviewer #3: Yes

**********

5. Is the manuscript presented in an intelligible fashion and written in standard English?

Reviewer #1: Yes

Reviewer #2: Yes

Reviewer #3: Yes

**********

Reviewer #1: I have reviewed the revised version of the manuscript titled "Synergistic Impact of ECAP and Thermal Treatments on the Corrosion Performance of AZ91 Mg Alloy." The authors have carefully addressed all of the reviewers' comments, including revisions to the experimental data, improvements in figure clarity, and a more thorough discussion of the experimental results. The revised manuscript is now better, with clearer explanations of the novelty, the synergistic effects of ECAP and heat treatment, and the statistical validation of the data.

After reviewing the manuscript and the authors' responses, I am pleased to inform you that we are satisfied with the revisions. Therefore, I would like to formally accept the manuscript for publication.

Reviewer #2: Dear Authors, I have reviewed the revised manuscript and find it significantly improved compared to the previous version. You have addressed all my previous comments clearly and satisfactorily. I recommend the paper for publication in its current form.

Reviewer #3: Please check the attached document

**********

what does this mean?). If published, this will include your full peer review and any attached files.

If you choose “no”, your identity will remain anonymous but your review may still be made public.

**Do you want your identity to be public for this peer review?**  For information about this choice, including consent withdrawal, please see our Privacy Policy

Reviewer #1: No

Reviewer #2: No

Reviewer #3: No

**********

[NOTE: If reviewer comments were submitted as an attachment file, they will be attached to this email and accessible via the submission site. Please log into your account, locate the manuscript record, and check for the action link "View Attachments". If this link does not appear, there are no attachment files.]

To ensure your figures meet our technical requirements, please review our figure guidelines: https://journals.plos.org/plosone/s/figures

You may also use PLOS’s free figure tool, NAAS, to help you prepare publication quality figures: https://journals.plos.org/plosone/s/figures#loc-tools-for-figure-preparation.

NAAS will assess whether your figures meet our technical requirements by comparing each figure against our figure specifications.

Attachment Submitted filename: Review Comments.docx

Attachment Submitted filename: PONE-D-25-66592_R1-review.pdf

# Author comment: Author response to Decision Letter 2

**DOI:** 10.1371/journal.pone.0354359.r004

23 Jun 2026

Review on the revised version of “Synergistic Impact of ECAP and Thermal Treatments on the

Corrosion Performance of AZ91 Mg Alloy”

The authors have updated the content to improve the article based on the recommendation of

various referees. While the work is interesting for PLOS One, there is still scope to improve and

clarify details before publication. The referee recommends the work to be considered for

publication after the following minor revision.

Here are some of the points the authors should address for future version:

1. Please provide the quantitative values for grain sizes related to Figure 1 for better

understanding in Section 3.1.

Response: revised in to text.

2. Figure 2 still contains a lot of information which is unclear. It will be great if the authors

could add a supplementary section and provide high resolution individual images where

the different aspects highlighted in the images are completely clear. The authors have

labeled α-Mg, Petal Shape, Eutectic Phase, Discontinuous distribution of secondary

phase. This is true for any other images shown later where the authors have labeled

different phases or features to be added in the supplementary section for clearer

reference.

Response: These SEM micrographs were obtained using the same imaging parameters for

consistent comparison under different process conditions. The labeled features like the α-Mg

matrix, petal-like morphology, eutectic formations and the discontinuous β-Mg17Al12 secondary

phases were determined through repeated observations via SEM, together with morphological

identification and further confirmed through EDS results and related literature. Despite the fact

that some of the finer features could be difficult to see at a particular comparative scale, these

micrographs provide sufficient evidence regarding the microstructural phenomena discussed in

the manuscript.

3. It is unclear what type of imaging is used for Figure 3 (Optical microscopy is mentioned

in part of the text). Please add it in the caption and in the text. Also, include the grain

sizes in the figure caption.

Response: we revised in to text.

4. Please refer to Tables 1 and 2 wherever necessary in the results section to guide the

readers on the original contribution better. For example, in the discussion of Figure 3, the

authors can refer to Table 2 for grain size measurement.

Response: we agree with you, we revised in to text.

5. Page 9, Section 3.3, 1st Paragraph, Line 4: sentence referring to Figure 4 for hear-treated

as-received alloy. Figure 4 does not have any as-received alloy micrographs. Please

update as needed.

Response: we revised in to text

The microstructures of AZ91 magnesium alloy subjected to two-pass (ECAP-2P) and four-pass

(ECAP-4P) processing, followed by heat treatments at 523 K, 623 K, and 723 K for durations

of 6 and 12 hours, are presented in Figures 4 and 5, respectively. In comparison to the heat

treated as-received alloy (Figure 2), the ECAP-processed specimens display a noticeably more

uniform and refined distribution of secondary phases across all annealing temperatures and

holding times…………..

6. Similar to the previous version, the authors are consistently indicating some phases are

better corrosion resistant without the corrosion data. It feels like, it would be better to

first show the corrosion data and then present the microscopies so that the text will have

more meaning. For example, in Section 3.3, Page 10, 1st Paragraph, last sentence clearly

concludes corrosion related performance by only referring to the micrographs, which is

confusing.

Response: we revised in to text.

7. In Page 10, Section 3.5, 1st Paragraph, the authors mentioned “the white corrosion

products”. Is there a chemical composition that can be referred here?

Response: In the present study, the “white corrosion products” observed on the surface of the

corroded AZ91 magnesium alloy primarily correspond to magnesium hydroxide (Mg(OH)_2_),

which forms due to the reaction of magnesium with the aqueous chloride-containing

environment. Included in the manuscript.

8. Based on the images shown in Figure 8, the as-received sample shows more corrosion

than the annealed samples, is that right? If this is correct, then in the text Page 15, Section

3.5, 2nd Paragraph, first sentence indicates that Figures 8b) and c) reveal more extensive

corrosion compared to the as-received alloy needs to be updated.

Response: revised in to text

9. Please re plot Figures 7, 9 and 10 to make it clearer. One way to do it is to keep the

symbols the same for the same temperature and use a filled symbol for 6 h and open

symbol for 12 h or vice versa. Also. the lines used to join the data points sometimes look

unnatural. For example, the 623 K – 6 h data shown in Figure 9

Response: revised in to text.

10. Page 17, 2nd Paragraph, Line 1: the authors mentioned “….lowest values of Icorr (0.0110

mA/cm² and 0.0011 mA/cm²) were….”. One of these current values do not match the

data in the Table. Please correct.

Response: Revised in to text, we thank the reviewer for highlighting this error, which has helped

improve the accuracy and consistency of the manuscript

11. Please move Figure 11 to the supplementary information as it is redundant. The data is

well presented in Table 1.

Response: To avoid redundancy and improve clarity, the manuscript has been revised to ensure that

Figure 11 is explicitly discussed as a visual interpretation of the quantitative trends summarized in

Table 1, thereby making both representations complementary rather than repetitive.

12. Page 21, the paragraph starting with “Although the current research….” Is this talking

about the work of the current article? Please clarify. This complete paragraph looks

disconnected.

Response: This pertains to the current study, but it is mentioned as a scope for future work.

13. What is the error bar of the data presented in Table 2? Please give a range or standard

deviation to understand the spread in grain sizes.

Response: revised in to text

14. There are some interesting patterns in Table 1 that the authors should indicate or discuss.

For example, why Ecorr of ECAP-4P gets worse from Room temperature to 523 K 6 h and

523 K 12 h, before getting better for 623 K onwards.

Response: revised in to text

Here are some minor corrections to implement:

1. Page 2, Introduction, 2nd Paragraph, Line 1: Recent investigations indicate….”. Please

refer some of the recent articles for supporting this sentence.

Response: included as per reviewer comments

2. Page 3, Introduction, 2nd Paragraph, Line 5: “Recent efforts in the field….”. It referes to

an article from 2021 [Ref 11]. This is not regarded as recent.

Response: revised

3. Page 3, Last Paragraph. Line 3: Replace “severe plastic deformation” with SPD.

Response: we revised in to text.

4. Page 4, Section 2, 1st Paragraph: font size of last few lines are smaller than the rest.

Response: revised.

5. Page 5, Line 3: EDS is used for the first time. Please use the full form here rather than

later (done in Page 5, Line before Equation 1)

Response: revised

6. Page 7, Section 3.2, 1st Paragraph, Line 5: Fix the formatting of β-Mg17Al12

Response: revised in to text.

7. Page 10, 1st Paragraph has most of the information in the beginning covered in the first

two paragraphs of Section 3.3 in Page 9. Please remove any redundancy.

Response: revised in to text.

8. Page 10, Last Line: Rewrite “shown in Table 2”

Response: revised in to text.

9. Page 16, Last Paragraph: “two passes (ECAP-2P) and four passes (ECAP-4P)” Please use

the acronym once mentioned earlier.

Response:

revised in to text.

10. Page 20, 2nd Paragraph: Fix the formatting of β-Mg17Al12 and Mg(OH)2.

Response: revised

11. Page 20: Please use ECAP, which is well established before rather than Equal Channel

Angular Pressing

Response: revised in to text.

12. Page 20, Last Paragraph, 1st Sentence needs reference. Without it, the second sentence

“ln this study, potentiodynamic….” becomes confusing. It feels like it is the work of the

current authors.

Response: revised in to text.

13. Please check the header formatting of Table 1, say Annealing Time (h) is wrapped in a

confusing way.

Response: revised

Review on “Synergistic Impact of ECAP and Thermal Treatments on the Corrosion

Performance of AZ91 Mg Alloy”

The authors have attempted to systematically investigate the mechanical and corrosion

behavior of AZ91 Mg alloy by performing ECAP 2P, 4P and heat treatment at various

temperatures and time. While the work is interesting for PLOS One, there is a lot of

scientific issues that are unclear and needs clarification before consideration for

publication. The referee recommends the work to be considered for publication

after major revision.

Here are some of the points the authors must address for future consideration:

1. Please guide the readers by clearly marking the secondary phases, eutectic phases,

residual phases or any other phases in a microstructure before describing the details.

Response: revised and In the microstructural images, the α-Mg matrix, eutectic structures,

residual β-Mg_1_₇Al_1__2_ phases, and redistributed secondary phases are explicitly marked.

2. In many instances, the authors have mentioned that the corrosion resistance is improved

in Section 3 without any supporting data. Please conclude only if you have supporting

evidence or wait till the evidence is shown to conclude on corrosion resistance.

Response: revised in to text

3. Figure 1 shows the SEM image of the as-received, 2P and 4P microstructures. While the

as-received microstructure is shown at x1000, the other two are shown at a higher

magnification. It is generally better to show the same magnification is available. While

the scale bar should be used to analyze the images independently, since these are shown

side-by-side, a quick glance may indicate that the grain sizes are the similar for all cases.

If the authors want to keep the same images, please include an average ASTM grain size

calculated for all the three cases both in the Figure caption and in the text for comparison.

This will support the conclusion in the last Paragraph of Page 5 on grain refinement due

to 2P and 4P with quantitative numbers.

Response: revised in to text

4. In Figure 1(a), the claim of coarse-grained structure is accepted, but it is better to

quantify it with the comment in 1. In addition, the claim that there are high fraction of

secondary phases along the grain boundaries are not clear at all. Please highlight the areas

or provide supporting evidence of this grain boundary segregation.

Response: revised in to text

5. In Page 5, last paragraph, the authors have reported that ECAP generates high density of

dislocations and activates dynamic recrystallization. There is no supporting data shown

for this conclusion. Please include the images showing dislocations or include references

to support this claim.

Response: revised in to text

6. The conclusions reported in the last Paragraph of Section 3.1 (Page 6) is very confusing.

It is unclear how one can assign shear planes, basal texture, etc. Please clarify.

Response: As no direct supporting experimental evidence was available to substantiate this

claim, the related discussion has been removed from the revised manuscript to avoid

overinterpretation

7. Page 6, Section 3.2, 1st Paragraph: It is unclear how to compare the secondary phases in

all the heat treated microstructures. Looking at the as-received one in Figure 1(a) with

respect to the ones in Figure 2, please highlight which is a secondary phase, how is the

eutectic structure identified in these images? What is the eutectic structure is also not

mentioned appropriately. Please guide the reader so that it is easy to understand the impact of

heat treatment in these alloys.

Response: revised

8. From Figure 2, it is also unclear on how much refinement is done with different

temperatures mentioned in the text. It is also unclear how the continuity of the secondary

phases are reported here by looking at a 2D image.

Response: revised in to text

9. There is some grain size measurement reported in Section 3.2 with heat treatment.

However, the numbers reported does not seem to match at least the as-received sample

microstructure shown in Figure 1(a). It looks like the grain size of Figure 1(a) should be

between 20 - 35 µm. How did the authors get a 58.69 µm for the as-received sample in

Figure 3(a). Please keep the data consistent over the various portion of the discussion.

Kindly clarify.

Response: The average grain size values were determined using quantitative image analysis

following an ASTM/intercept-based approach from multiple representative micrographs,

rather than visual estimation from a single image, to ensure statistical reliability and

consistency

The grain size value of 58.69 µm was determined from OM images after metallographic

etching, which provides clearer grain boundary delineation than SEM images

10. In Page 10, 1st Paragraph, the authors have mentioned reduction of residual phases. What

are these? Kindly define every possible phase that are discussed in a better way so that

the audience can keep track of all the changes in the microstructure based on the

supporting evidence.

Response: revised in to text

11. Page 10, 2nd Paragraph also refers to the increased fractions of the β-phase without any

clear guidance from the SEM images shown.

Response: revised

12. Why Figure 6 has a blue filter? Please include SEM in the caption.

Response: Figure 6. Optical Microscopy (OM) images showing the microstructural evolution of AZ91

magnesium alloy after different thermal treatment conditions. As it is OM images that’s why it is blue

filter.

13. The authors have reported the Mechanical properties in the last paragraph of Section 3.3.

It will be better to include the stress-strain diagram for these and mention the sample

preparation for these tests. Also, for hardness, it is necessary to include all the different

cases reported so far and not just as-received and one of the heat treated 4P samples.

Response: Thank you for this valuable suggestion. We agree that detailed mechanical

characterization, including stress–strain diagrams, sample preparation procedures, and

comprehensive hardness evaluation for all processing conditions, would further strengthen the

discussion.

However, these investigations were beyond the scope of the present study, which primarily

focuses on the microstructural evolution and corrosion behavior of ECAP-processed and heat

treated AZ91 magnesium alloy. We consider the inclusion of detailed mechanical

characterization as an important direction for future work

14. Figures 7, 9, and 10 needs to be improved. It is not very easy to see the trends based on

the way the data is plotted (with the symbols and lines combinations used for the plots).

Response: revised

15. In Page 14, the authors have referred to Equation (1) to compute Ecorr, Icorr, and Corrosion

Rate. However, Equation (1) is not reported in the text. This type of overlook is not

appropriate. Please correct.

Response: revised

Kindly update the recommended modifications and go through the corrosion data while

finalizing the article for re-evaluation. Here are some minor corrections to implement:

1. Page 2, Introduction, 1st Paragraph, Line 10: “…., contributing to enhanced strength and

ductility”. Please make sure both strength and ductility were enhanced at the same time

or if strength is enhanced ductility is decreased.

Response: revised

“…, contributing to enhanced mechanical performance through grain refinement and microstructural

modification, depending on the processing conditions.

Page 8, Introduction, 2nd Paragraph: starts with “Recent investigations indicate that ECAP….”.

This is supported by Reference [11], which is from 2021. This is not recent. Also,

“investigations” mean there has to be more than one article referred for that statement. Please

update.

Response: revised

2. Page 3, 2nd Paragraph, Line 7: Please rewrite “…significant international progress…”. It

is unclear what this means.

Response: revised

3. Page 4, Last Paragraph: Define “OM” before using. Is it optical microscope?

Response: revised

4. In Page 4, the authors have reported ECAP was performed at 325 °C, while in Page 5

Section 3.1, 1st Paragraph the authors have indicated the ECAP done at 598 K. This is

confusing. Please use the same temperature unit so that one do not need to convert from

one unit to the other.

Response: revised

Attachment Submitted filename: Answar_to_Reviewer_Comments_auresp_2.pdf

# Editor report: Decision Letter 2

**DOI:** 10.1371/journal.pone.0354359.r005

**Authors:** Kuldeep Kumar Saxena

7 Jul 2026

Synergistic Impact of ECAP and Thermal Treatments on the Corrosion Performance of AZ91 Mg Alloy

PONE-D-25-66592R2

Dear Dr. Anne,

We’re pleased to inform you that your manuscript has been judged scientifically suitable for publication and will be formally accepted for publication once it meets all outstanding technical requirements.

Within one week, you’ll receive an e-mail detailing the required amendments. When these have been addressed, you’ll receive a formal acceptance letter and your manuscript will be scheduled for publication.

An invoice will be generated when your article is formally accepted. Please note, if your institution has a publishing partnership with PLOS and your article meets the relevant criteria, all or part of your publication costs will be covered. Please make sure your user information is up-to-date by logging into Editorial Manager at Editorial Manager® and clicking the ‘Update My Information' link at the top of the page. For questions related to billing, please contact billing support.

If your institution or institutions have a press office, please notify them about your upcoming paper to help maximize its impact. If they’ll be preparing press materials, please inform our press team as soon as possible -- no later than 48 hours after receiving the formal acceptance. Your manuscript will remain under strict press embargo until 2 pm Eastern Time on the date of publication. For more information, please contact onepress@plos.org.

Kind regards,

Kuldeep Kumar Saxena

Academic Editor

PLOS One

Additional Editor Comments (optional):

Reviewers' comments:

# Editor report: Acceptance letter

**DOI:** 10.1371/journal.pone.0354359.r006

**Authors:** Kuldeep Kumar Saxena

PONE-D-25-66592R2

PLOS One

Dear Dr. Anne,

I'm pleased to inform you that your manuscript has been deemed suitable for publication in PLOS One. Congratulations! Your manuscript is now being handed over to our production team.

At this stage, our production department will prepare your paper for publication. This includes ensuring the following:

* All references, tables, and figures are properly cited

* All relevant supporting information is included in the manuscript submission,

* There are no issues that prevent the paper from being properly typeset

You will receive further instructions from the production team, including instructions on how to review your proof when it is ready. Please keep in mind that we are working through a large volume of accepted articles, so please give us a few days to review your paper and let you know the next and final steps.

Lastly, if your institution or institutions have a press office, please let them know about your upcoming paper now to help maximize its impact. If they'll be preparing press materials, please inform our press team within the next 48 hours. Your manuscript will remain under strict press embargo until 2 pm Eastern Time on the date of publication. For more information, please contact onepress@plos.org.

You will receive an invoice from PLOS for your publication fee after your manuscript has reached the completed accept phase. If you receive an email requesting payment before acceptance or for any other service, this may be a phishing scheme. Learn how to identify phishing emails and protect your accounts at https://explore.plos.org/phishing.

If we can help with anything else, please email us at customercare@plos.org.

Thank you for submitting your work to PLOS ONE and supporting open access.

Kind regards,

PLOS ONE Editorial Office Staff

on behalf of

Dr. Kuldeep Kumar Saxena

Academic Editor

PLOS One
